# Escape From Cisplatin-Induced Senescence of Hypoxic Lung Cancer Cells Can Be Overcome by Hydroxychloroquine

**DOI:** 10.3389/fonc.2021.738385

**Published:** 2022-01-21

**Authors:** Aleksandra Olszewska, Agata Borkowska, Monika Granica, Justyna Karolczak, Bartosz Zglinicki, Claudine Kieda, Halina Was

**Affiliations:** ^1^ Laboratory of Molecular Oncology and Innovative Therapies, Military Institute of Medicine, Warsaw, Poland; ^2^ Postgraduate School of Molecular Medicine, Medical University of Warsaw, Warsaw, Poland; ^3^ Doctoral School of Translational Medicine, Centre of Postgraduate Medical Education, Warsaw, Poland

**Keywords:** cancer, hypoxia, senescence, autophagy, chemoresistance

## Abstract

Chemotherapy is the commonly used treatment for advanced lung cancer. However, it produces side effects such as the development of chemoresistance. A possible responsible mechanism may be therapy-induced senescence (TIS). TIS cells display increased senescence-associated β-galactosidase (SA-β-gal) activity and irreversible growth arrest. However, recent data suggest that TIS cells can reactivate their proliferative potential and lead to cancer recurrence. Our previous study indicated that reactivation of proliferation by TIS cells might be related with autophagy modulation. However, exact relationship between both processes required further studies. Therefore, the aim of our study was to investigate the role of autophagy in the senescence-related chemoresistance of lung cancer cells. For this purpose, human and murine lung cancer cells were treated with two commonly used chemotherapeutics: cisplatin (CIS), which forms DNA adducts or docetaxel (DOC), a microtubule poison. Hypoxia, often overlooked in experimental settings, has been implicated as a mechanism responsible for a significant change in the response to treatment. Thus, cells were cultured under normoxic (~19% O_2_) or hypoxic (1% O_2_) conditions. Herein, we show that hypoxia increases resistance to CIS. Lung cancer cells cultured under hypoxic conditions escaped from CIS-induced senescence, displayed reduced SA-β-gal activity and a decreased percentage of cells in the G2/M phase of the cell cycle. In turn, hypoxia increased the proliferation of lung cancer cells and the proportion of cells proceeding to the G0/G1 phase. Further molecular analyses demonstrated that hypoxia inhibited the prosenescent p53/p21 signaling pathway and induced epithelial to mesenchymal transition in CIS-treated cancer cells. In cells treated with DOC, such effects were not observed. Of importance, pharmacological autophagy inhibitor, hydroxychloroquine (HCQ) was capable of overcoming short-term CIS-induced resistance of lung cancer cells in hypoxic conditions. Altogether, our data demonstrated that hypoxia favors cancer cell escape from CIS-induced senescence, what could be overcome by inhibition of autophagy with HCQ. Therefore, we propose that HCQ might be used to interfere with the ability of senescent cancer cells to repopulate following exposure to DNA-damaging agents. This effect, however, needs to be tested in a long-term perspective for preclinical and clinical applications.

## Introduction

Lung cancer remains the leading cause of cancer-related deaths. Approximately 85% of all lung cancer cases are nonsmall cell lung cancer (NSCLC) and adenocarcinoma is the most common histological subtype of NSCLC (1, 2). Despite the development of targeted therapies, platinum-based chemotherapy (CIS or carboplatin) is still a predominant treatment regimen for advanced lung cancer patients. In general, it is combined with other chemotherapeutic drugs: vinorelbine, gemcitabine, or taxanes (DOC or paclitaxel). Unfortunately, the treatment is not always effective. Stage III disease is correlated with poor prognosis, where the 5-year survival probability is only 15%. Moreover, patients who are subjected to such treatment, experience systemic toxicity and many adverse effects, including the following: hair and weight loss, vomiting, greater risk of infection, as well as development of secondary resistance and cancer recurrence (3–5). A responsible mechanism of action might be cellular senescence. This process can be induced by telomere shortening (replicative senescence) or by various stress factors [stress-induced premature senescence (SIPS)] (6). One of the most recently described SIPS is therapy-induced senescence (TIS) (7–10). Besides arrest of cell proliferation, hallmarks of cells displaying TIS are as follows: morphological changes (cell flattening and enlargement), enhanced activity of SA-β-gal, higher production of cytokines and chemokines described as senescence-associated secretory phenotype (SASP), and activation of the DNA damage response (DDR) pathway (5, 11, 12). The induction of senescence has been considered for decades as a satisfactory objective for positive treatment outcome (10, 13, 14). However, it has been largely documented that tumor heterogeneity, plasticity, and adaptation may cause TIS cells to escape from senescence and return into an actively reproductive state, leading to cancer recurrence (7, 15–17). A feature of TIS cells associated with the tumor repopulation might be polyploidy, which determines their ability for atypical divisions (18–21). In addition, the literature suggests that TIS may result in activation of cancer cell invasive properties and induction of stemness phenotype that may contribute to an increased risk of tumor relapse and metastasis (16, 17, 22–26). Therefore, the induction of senescence itself seems to be an insufficient endpoint and additional strategies are needed to achieve a better therapeutic response.

Prior studies, including ours, have indicated that chemotherapy-induced senescence is accompanied by autophagy. Therefore, modulation of this process seems to be an attractive therapeutic strategy for cancer (16, 27, 28). Autophagy is a catabolic process responsible for the recycling of cellular compounds, which allows cells to maintain cellular homeostasis and provides substrates for energy production (29). In cancer, autophagy and senescence show many, often overlapping activities, including an impact on tumor development and modulation of responses to chemotherapy (30). However, the exact relationship between autophagy and senescence is not clear (31–33), pointing to the need for further research that would consider the biologically relevant microenvironmental conditions of tumor growth and proper perspective of treatment intervals.

Hypoxia is a crucial variable in solid tumors, which should be considered in studies evaluating chemoresistance. Although, it is commonly overlooked in *in vitro* experiments. The examination of tumor cells under normoxic conditions brings an artificial context in terms of oxygen balance, which can be misleading to interpret cell actual response to therapies (34). Therefore the research in a hypoxic environment is closer to the conditions in the tumor compared to standard normoxic culture. Recent data on clinical samples confirm significant changes in the transcriptome of cancer cells in response to hypoxia and suggest their prognostic associations with drug response (35, 36). Importantly, resistance to chemotherapy caused by hypoxia has been reported in many types of cells and the presence of hypoxic sites is a prognostic factor of neoplastic diseases (37, 38). However, the role of senescence and autophagy in the chemoresistance of hypoxic cancer cells has not been studied in detail.

Thus, in this study, we investigated the effects of hypoxia on therapy-induced senescence of lung cancer cells. Additionally, we examined, whether inhibition of autophagy using the autophagy inhibitor hydroxychloroquine (HCQ) could modulate the ability of TIS cells to escape senescence.

## Materials and Methods

### Reagents and Antibodies

CIS, DOC, irinotecan (IRINO), HCQ, and 3-(4,5-dimethylthiazol-2-yl)-2,5-diphenyltetrazolium bromide (MTT) were obtained from Sigma Aldrich, St. Louis, MO, USA. Nutlin-3 (NUT) was obtained from Tocris Bioscience, Bristol, UK. Antibodies against p53 and p16 were purchased from Santa Cruz Biotechnology, Dallas, TX, USA. Antibodies against: E-cadherin, N-cadherin, cyclin B, p-Rb, p-cdc2, SOX-2, and Nanog were procured from Cell Signaling Technology, Danvers, MA, USA. Antibodies against p21 were bought from Sigma Aldrich, St. Louis, MO, USA. Antibodies against p-S6 (Ser235/236) and p62 were purchased from BD Transduction Laboratories™, Lexington, KY, USA. Antibodies against GAPDH were procured from Millipore, Temecula, CA, USA and ALDH1A from LifeSpan Biosciences, Seattle, WA, USA. Secondary antimouse and antirabbit antibodies conjugated with HRP were obtained from Vector Laboratories, Burlingame, CA, USA.

### Cells and Culture Conditions

The human A549 lung cancer cell line was kindly provided by Prof. Bozena Kaminska-Kaczmarek (Nencki Institute of Experimental Biology, Warsaw, Poland). The human colon HCT116 cancer cell line was kindly provided by Dr. Bert Vogelstein (Johns Hopkins University, Baltimore, MD, USA). Authentication of these cells was performed by the American Type Culture Collection (ATCC). The LLC1 murine Lewis lung carcinoma cell line was purchased from the ATCC. A549 and LLC1 cells were cultured in high-glucose Dulbecco’s modified Eagle’s medium (Gibco, Life Technologies Limited, Middlesex, UK) supplemented with 10% fetal bovine serum (Gibco, Life Technologies Limited, UK), while HCT116 in McCoy’s medium supplemented with 10% fetal bovine serum (Bio-West, Nuaillé, France). Both media were supplemented with: 100 units/ml of penicillin, 100 μg/ml of streptomycin, and 25 μg/ml of amphotericin B (Antibiotic-Antimycotic, Gibco, Life Technologies Corporation, Grand Island, NY, USA). All experiments were performed in normoxia (~19% O_2_) or hypoxia (1% O_2_) under standard culture conditions (37°C, 5% CO_2_). After experiments, cells were collected by trypsinization (Gibco, Life Technologies Limited, UK). Hypoxic phenotype was confirmed using NGS method. Expression of hypoxia-induced mir210HG and Egl-9 Family Hypoxia Inducible Factor 3 (EGLN3), for example, was elevated in cells cultured under hypoxic conditions (data not shown).

### General Experimental Scheme

In the experiments performed on 96-well plates, cells were seeded at 1,500 cells/per well for A549 cells and 91 cells per well for LLC1 cells. For the remaining tests, 125,000 cells were plated on 25 cm^2^ flask for each A549, LLC1, or HCT116 cell line. After seeding, cells were grown in normoxia for 24 h. Some plates/flasks were then left in normoxic condition, whereas other culture dishes were transferred to the hypoxic chamber, and medium was changed. The hypoxic medium was incubated for at least for 24 h in the hypoxic chamber prior to the experimental procedure. After 24 h of culture in normoxia or hypoxia, cells were treated with chemotherapeutics for the next 24 h. CIS was used at a concentration of 8 µM for A549 or 6 µM for LLC1 cells, and DOC was used at 3 nM and IRINO at 5 µM concentration. After 24 h incubation with chemotherapeutic drugs, medium was changed and cells were cultured in drug-free medium for the next 7 days. Additionally, medium was changed on day 4.

### Activation of p53 With Nutlin-3

To study the role of p53 in escaping of hypoxic CIS-treated cells from senescence, nutlin-3 (NUT) was applied together with CIS. After 24 h, medium was changed and cells were cultured in drug-free medium for the next 7 days. Additionally, medium was changed on day 4.

### Inhibition of Autophagy by Hydroxychloroquine

Experiments with hydroxychloroquine (HCQ) were carried out as described above (**General Experimental Scheme**) up to day 4 after chemotherapeutics treatment. At that point, to inhibit autophagy, HCQ was administered for 24 h. Subsequently, the medium was changed and cells were cultured in drug-free medium for the next 3 days. For gene expression analysis, cells were collected on day 8 of the experiment. For all other analyses, cells were collected on day 11.

### Silencing of Autophagy-Related Genes Using siRNAs

A549 cells were seeded at 750 per well or 62,500 cells per 25 cm^2^ flask in normoxic conditions. Twenty-four hours later, some plates/flasks were left in normoxic condition, whereas other culture dishes were transferred to the hypoxic chamber and the medium was changed. After a 24-h incubation, cells were transfected with pooled siRNAs targeting autophagy-related genes: ATG5, ATG7, or BCN1 (ON-TARGETplus siRNA, Dharmacon, Lafayette, CO, USA) using lipofectamine RNAiMAX (Invitrogen, Life Technologies Corporation, Carlsbad, CA, USA). Nontargeting siRNAs was used as a control. Lipofectamine and siRNA dilutions were prepared according to manufacturers’ protocols. Next day, cancer cells were treated with respective drugs for 24 h followed by 7 days of drug-free culture. All analyses were performed on day 12 of the experiment.

### Western Blotting

Cells were lysed in RIPA lysis buffer (Thermo Scientific, Rockford, IL, USA) and then lysates were frozen. After defrosting, the lysates were centrifuged at 10,000×*g*. The protein concentration was determined using the Pierce BCA Protein Assay Kit (Thermo Scientific, Rockford, IL, USA), and aliquots were harvested into Laemmli SDS sample lysis buffer (Bio-Rad, Hercules, CA, USA or EurX, Gdańsk, Poland). The same protein amount (10–50 μg) was loaded into wells for separation by SDS-PAGE electrophoresis and then transferred to a nitrocellulose membrane (Bio-Rad, Feldkirchen, Germany). After proteins were transferred, the membrane was blocked with 5% nonfat milk in Tris-buffered saline pH 7.6/0.15% Tween 20 (TBST) for 1 h at room temperature (RT) and then incubated with primary antibodies diluted in a TBST overnight at 4°C. Next, the membrane was incubated for 1 h at RT with relevant secondary antibodies. Protein signals were visualized on X-ray film with the use of chemiluminescent substrates (Santa Cruz, Santa Cruz, CA, USA or Bio-Rad, Segrate, Italy). The protein size was determined using a protein size-marker (Thermo Fisher Scientific, Waltham, MA, USA). Densitometric analysis was carried out using ImageJ software.

### Hypertrophy Evaluation

Cellular hypertrophy was calculated as the amount of total protein and normalized to total cell number. Protein concentration was examined using the BCA method (see **Western Blotting**). Total cell number was counted using Bürker’s chamber. The results were presented as the amount of total protein per cell.

### Measurement of Mitochondrial Dehydrogenase Activity

Mitochondrial dehydrogenase activity was evaluated by measuring the conversion of MTT (final concentration of 0.5 mg/ml) to formazan crystals in living cells. Cells were cultured in 96-well plates as described previously. Seven days after treatment with drugs, cells were incubated for 2 h with MTT at 37°C. Obtained crystals were then dissolved in 50 mM HCl diluted with isopropanol. The optical density was measured at a wavelength of 562 nm using a scanning multiwell Varioskan Lux spectrophotometer (Thermo Scientific).

### Analysis of Cell Proliferation

Proliferation of lung cancer cells was performed using the BrdU incorporation assay (Roche, Mannheim, Germany). Cells were cultured in 96-well plates as described previously. Seven days after treatment with drugs, BrdU was added for 2 h, and its incorporation was evaluated according to the manufacturer’s instructions. The optical density was measured at a wavelength of 450 nm using a scanning multiwell Varioskan Lux spectrophotometer (Thermo Scientific).

### Detection of SA-β-Gal

SA-β-Gal activity was detected according to modified protocol described by Dimri et al. (39). Cells were trypsinized, fixed with 2% formaldehyde, 0.2% glutaraldehyde in PBS, and washed with PBS. Next, 80,000 cells were cytospined at 800 rpm for 5 min and exposed to a solution containing: 1 mg/ml 5-bromo-4-chloro-3-indolyl-b-d-galactopyranoside, 5 mM potassium ferrocyanide, 150 mM NaCl, 2 mM MgCl_2_, and 0.1 M phosphate buffer, pH 6.0 for 16 h in 37°C. Microscopic slides were then washed and sealed with fluorescence mounting medium (Dako, Carpinteria, CA, USA). SA-β-Gal-positive and SA-β-Gal-negative cells were calculated (at least 100 cells per condition). Results were presented as a percentage of SA-β-Gal-positive cells.

### Flow Cytometry Analysis of Cell Cycle

For DNA analysis, live and adherent cells were collected by trypsinization, centrifuged at 1,200×*g* for 5 min, suspended in 500 μl PBS, and fixed in frozen 70% ethanol. Fixed cells were stored at −20°C. Before analysis, cells were washed with PBS, centrifuged, and suspended in 200 μl of Muse™ Cell Cycle Reagent (Millipore, Hayward, CA, USA) with propidium iodide (PI). Histograms of cellular DNA content were collected with Beckman Coulter CytoFLEX flow cytometer (Brea, CA, USA). FSC/SSC parameter and DNA content analysis were performed using CytExpert software. Detailed analysis was performed as follows. Events considered as cell debris were excluded from analysis using the FSC/SSC parameters. Subsequently, on the basis of the PI fluorescence intensity, the phases of the cell cycle were identified: subG1 (before the first peak), G0/G1 (first peak), S (between the first and second peaks), G2/M (second peak), and polyploidy (after the second peak). Results were showed as percentage of cells accumulated in the distinct cell cycle phases. At least 10,000 events were analyzed for each sample.

### RNA Extraction and qPCR

RNA was extracted using RNeasy Mini Kit (Qiagen, Hilden, Germany) following the manufacturer’s instructions. The concentration and quality of RNA were evaluated using a Varioskan Lux spectrophotometer (Thermo Scientific). Next, the extracted mRNA was subjected to reverse transcription using the High-Capacity cDNA Reverse Transcription Kit (Applied Biosystems, Thermo Fisher Scientific Baltics, UAB, Vilnius, Lithuania). The cDNA obtained was used as a template in the qPCR reaction. For HIF and E-cadherin genes, SsoAdvanced Universal SYBR Green Supermix (Bio-Rad, Hercules, CA, USA) was used, GAPDH served as a reference, and the following human primer sets were used: hypoxia inducible factor 1α (HIF-1α): for-ATTTTGGCAGCAACGACACA and rev-CGTTTCAGCGGTGGGTAATG, hypoxia inducible factor 2α (HIF-2α): for-CACCTCGGACCTTCACCAC and rev-GCTACTCCTTTTCTTCTCCTTGT, E-cadherin: for-CTTTGACGCCGAGAGCTACA and rev-AAATTCACTCTGCCCAGGACG, and GAPDH: for-GGAGCGAGATCCCTCCAAAAT and rev-GGCTGTTGTCATACTTCTCATGG. For other genes, the TaqMan Gene Expression Master Mix (Applied Biosystems, Thermo Fisher Scientific Baltics, UAB, Lithuania) was used, PPIA served as a reference gene, and following primers were obtained from Thermo Scientific: SOX-2, Hs01053049_s1; ALDH1A, Hs00946916_m1; Nanog, Hs04399610_g1; OCT-4, Hs04260367_gH; and PPIA, Hs01565699_g1.

### Statistical Analysis

All experiments were performed at least three times. Results were expressed as mean ± SEM. *p*-values were calculated in GraphPad PRISM v.9.0. and *p* -values < 0.05 were recognized as statistically significant (*vs. N control; #vs. H control, $ N vs. H, % 8 vs. 11). The normality of the distributions of numerical data was checked using the Shapiro-Wilk test. For normal distribution, groups were compared using a two-tailed unpaired *t*-test with Welch’s correction. In the case of an abnormal distribution, a nonparametric two-tailed Mann-Whitney *U* test was used.

## Results

### Lung Cancer Cell Cultures Showed Higher Resistance to Cisplatin in Hypoxic Than in Normoxic Conditions

**Figure 1:**
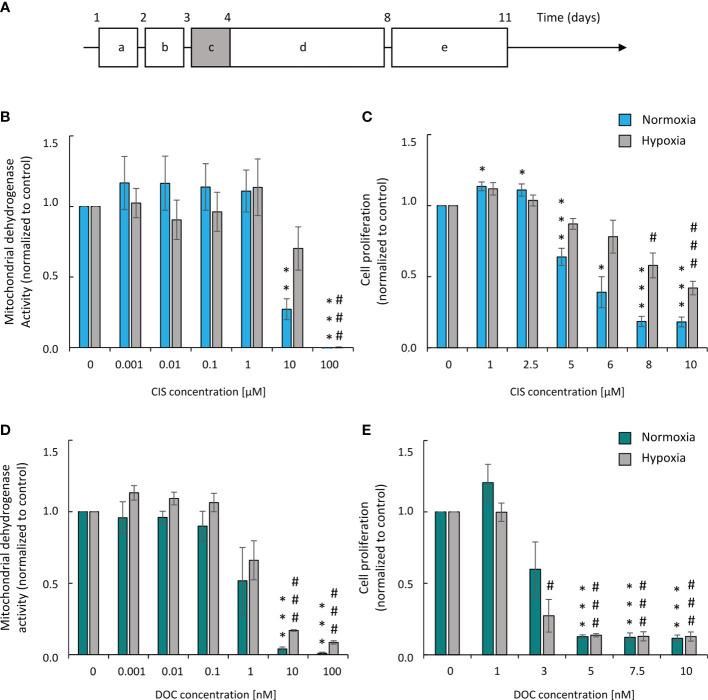
Human A549 lung cancer cells show higher resistance to CIS in hypoxic than in normoxic conditions. Experimental scheme **(A)**. Cells were grown in normoxic conditions (~19% O2) for 24 hours **(a)**. Then, some plates or flasks were transferred to the hypoxic chamber and the medium was changed **(b)**. After 24 hours, cells in the normoxia or hypoxia conditions were treated with chemotherapeutics for 24 hours **(c)** and then they were cultured in drug-free medium for 4 days **(d)**. Next medium was changed and cells were incubated next 3 days **(e)**. Analysis and quantification of cell metabolism and proliferation was performed on 11th day of the experiment (according to scheme A). Mitochondrial dehydrogenase activity (MDH) test in normoxia or hypoxia **(B)** was performed for assessment of CIS effect on cell metabolic activity. BrdU incorporation test in normoxia or hypoxia **(C)** was performed for assessment of CIS effect on cell proliferation. Both tests were conducted also for DOC-treated cells: MDH in normoxia or hypoxia **(D)** and BrdU assay in normoxia or hypoxia **(E)**. Each bar represents mean ± SEM. The respective P-values were calculated using a two-tailed t test and a P-value < 0.05 was considered statistically significant: *P < 0.05, **P < 0.01, ***P < 0.001 comparing to normoxic control, ^#^P < 0.05, ^##^P < 0.01, ^###^P < 0.001 comparing to hypoxic control, n ≥ 3.

A two-drug regimen based on a platinum derivative and a drug disrupting the microtubule function is most often used in patients with lung cancer. Therefore, we tested the effects of two agents from these groups: CIS and DOC on the induction of senescence of lung cancer cells *in vitro*. To mimic a regime of chemotherapy in patients, we subjected human lung cancer A549 cell cultures to long-term treatment with a chemotherapeutic drug. The cells were treated with CIS or DOC for 24 h. After drug removal, cells were cultured in a drug-free medium for the next 7 days (**Figure 1A**). To reproduce the cell response to chemotherapy in the natural microenvironment, we also investigated the effects of both chemotherapeutic agents under hypoxic conditions. The aim of the initial experiments was to define the senescence-inducing dose of CIS and DOC. They were identified as doses that were subtoxic (**Figures 1B, D**), inhibited cell proliferation (**Figures 1C, E**), and produced morphological changes: increased size and granularity as well as polyploidization (data not shown). Under CIS, the mitochondrial dehydrogenase activity of A549 lung cancer cells was moderately decreased under hypoxic conditions compared with the normoxic condition (**Figure 1B**). Accordingly, the effects on A549 cell proliferation were less pronounced under hypoxia than in normoxia (**Figure 1C**). In contrast, such differences in cell responses to hypoxia vs. normoxia were not observed, when cells were treated with DOC (**Figures 1D, E**). On the basis of above tests, the senescence-inducing doses of drugs for further experiments were selected as 8 µM for CIS and 3 nM for DOC. Analogous analyses were performed for the murine LLC1 cell line and the 6-μM dose of CIS was chosen (**Supplementary Figures 1A, B**).

Altogether, the data demonstrated that A549 lung cancer cell cultures exhibited a higher resistance to cisplatin in hypoxic than in normoxic conditions, while DOC treatment did not induce similar effects.

### Upon Hypoxia Human and Murine Lung Cancer Cells Escaped From Cisplatin-Induced Senescence

**Figure 2:**
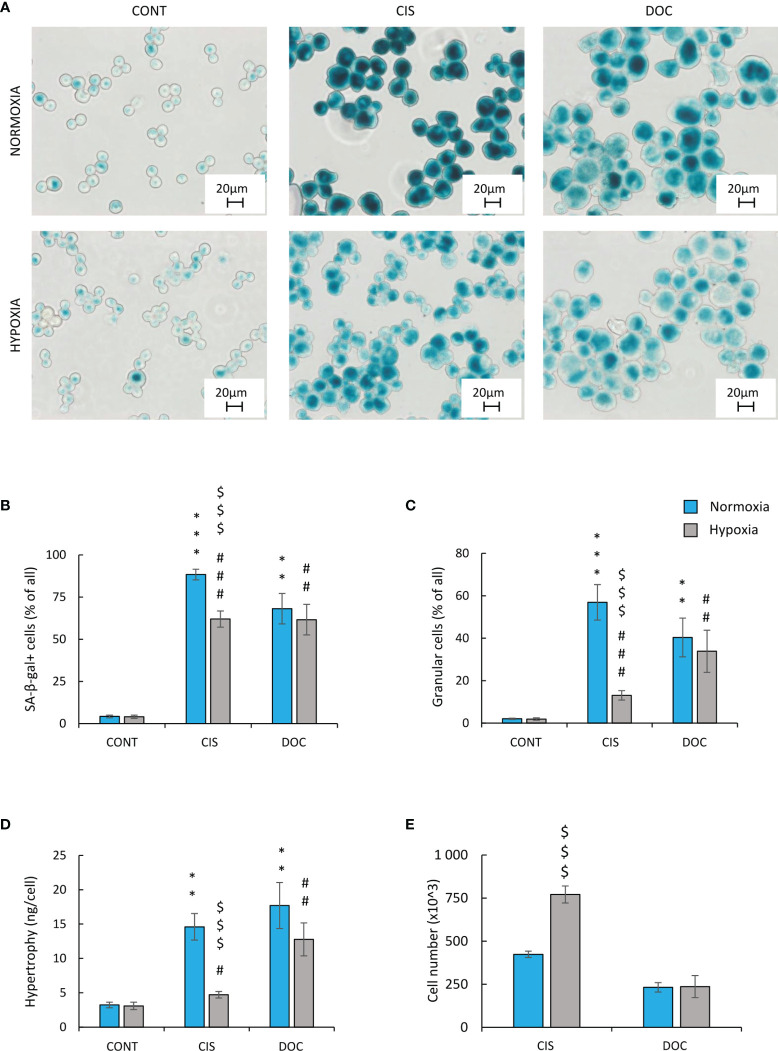
Hypoxia reduces markers of senescence and increases proliferation of CIS-treated human lung cancer cells. Detection and quantification of senescence and proliferation markers in cells after CIS or DOC treatment were performed on the 11th day of the experiment (according to **Figure 1A**). Detection and quantification of senescence were conducted using SA-β-gal staining on cytospined A549 lung cancer cells. Representative photos were acquired using light microscopy: original magnification, ×400; scale bar, 20 µm **(A)**. Quantification of SA-β-gal-positive cells shown as a percentage of positive cells **(B)**. Percentages of granular cells were determined by FSC/SSC analysis using flow cytometry **(C)**. Hypertrophy was defined as the ratio of the amount of total protein measured by BCA method to the number of A549 cells counted using Bürker’s chamber **(D)**. Evaluation of cell number after chemotherapeutics treatment was performed using Bürker’s chamber **(E)**. Each bar represents mean ± SEM. The respective *p*-values were calculated using a two-tailed *t*-test or Mann-Whitney *U* test, and a *p-*value <0.05 was considered statistically significant: ^*^ *p* *<* 0.05, ^**^ *p* *<* 0.01, ^***^ *p* *<* 0.001 comparing with normoxic control, ^#^ *p* *<* 0.05, ^##^ *p* *<* 0.01, ^###^ *p* *<* 0.001 comparing with hypoxic control, ^$^ *p* *<* 0.05, ^$$^ *p* *<* 0.01, ^$$$^ *p* *<* 0.001 comparing hypoxia with normoxia, *n* ≥ 3.

**Figure 3:**
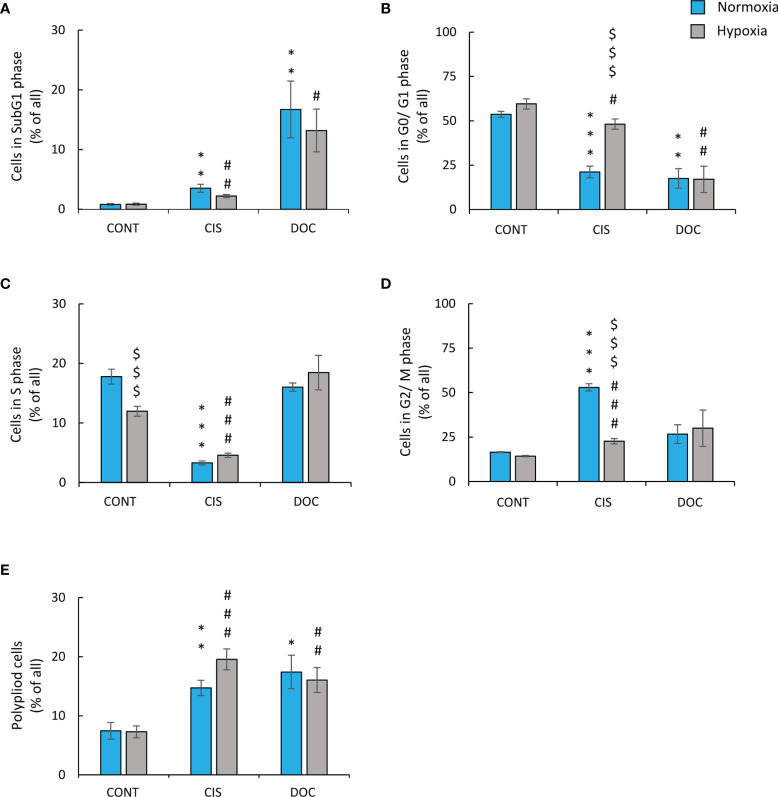
Low oxygen level modifies distribution of CIS-treated human lung cancer cells in the cell cycle. Analysis and quantification of cell cycle distribution was performed on the 11th day of the experiment (according to **Figure 1A**). Cell cycle distribution after CIS or DOC treatment was performed using PI staining and flow cytometry: sub-G1 **(A)**, G0/G1 **(B)**, S **(C)**, G2/M **(D)**, and polyploidy **(E)**. The respective *p*-values were calculated using a two-tailed *t*-test or Mann-Whitney *U* test, and a *p*-value <0.05 was considered statistically significant: ^*^ *p* *<* 0.05, ^**^ *p* *<* 0.01, ^***^ *p* *<* 0.001 comparing with normoxic control, ^#^ *p* *<* 0.05, ^##^ *p* *<* 0.01, ^###^ *p* *<* 0.001 comparing with hypoxic control, ^$^ *p* *<* 0.05, ^$$^ *p* *<* 0.01, ^$$$^ *p* *<* 0.001 comparing hypoxia with normoxia, *n* ≥ 3.

To test the hypothesis of the effects of hypoxia on senescence escape, several markers were investigated. Seven days after CIS treatment, the cells displayed an increased expression of senescence markers: SA-β-gal activity counted as a percentage of positive cells (**Figures 2A, B**), granularity (**Figure 2C**), or hypertrophy expressed as the amount of total protein (**Figure 2D**). Of note, all studied markers showed lower expression, when cells were treated with CIS in hypoxic conditions in comparison with normoxic ones (**Figures 2A**–**D**). Consequently, in hypoxia, 7 days after chemotherapeutics treatment, a significant increase in the cell number was observed, indicating cell proliferation and escape from senescence (**Figure 2E**). A similar pattern of changes was observed for murine LLC1 lung cancer cells (**Supplementary Figures S1C**–**E**). It is worth noting, that in the case of cells treated with DOC, no difference in the expression of senescence (**Figures 2A**–**D**) or proliferation (**Figure 2E**) markers was observed between the distinct aerobic conditions. As we have previously shown for the colon and glioma cancer cells, escape from senescence is associated with significant changes in the cell cycle distribution (16). In normoxia, 7 days after CIS treatment, lung cancer cells escaped from the G0/G1 phase (**Figure 3B**) and accumulated markedly in the G2/M phase (**Figure 3D**) with a reduced accumulation in S phase (**Figure 3C**) and an increased polyploidization (**Figure 3E**). In contrast, hypoxic cells escaped from G2/M (**Figure 3D**) and proceeded to the G0/G1 phase (**Figure 3B**), which suggests active progression through the cell cycle. However, hypoxia did not produce a significant effect on the distribution of cells in the S phase (**Figure 3C**) or on polyploidization (**Figure 3E**). Similarly, to CIS treatment in normoxia, DOC treatment downregulated the percentage of cells in the G0/G1 phase (**Figure 3B**) and augmented polyploidization (**Figure 3E**). Additionally, there was almost a tenfold increase in the percentage of cells in the sub-G1 phase after DOC treatment (**Figure 3A**), which indicated a significant proportion of cell death. As previously described, there were no observed effects due to hypoxia (**Figures 3A**–**E**).

**Figure 4:**
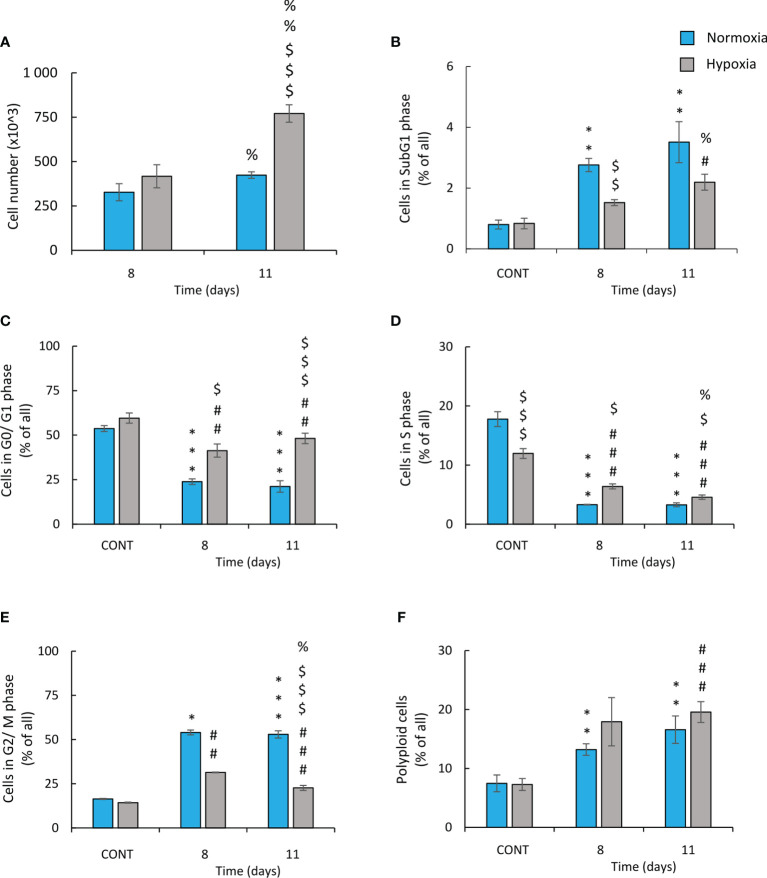
Hypoxic human lung cancer cells treated with cisplatin escape from G2/M phase and increase proliferation. Cell number and cell cycle distribution after CIS treatment was assessed on the 8^th^ or 11^th^ day of the experiment (according to **Figure 1A**). Quantification of cell number after CIS treatment was performed using Bürker’s chamber **(A)**. Cell cycle distribution was conducted using PI staining and flow cytometry: sub-G1 **(B)**, G0/G1 **(C)**, S **(D)**, G2/M **(E)**, and polyploidy **(F)**. The respective *p*-values were calculated using a two-tailed *t*-test or Mann-Whitney *U* test and, a *p*-value <0.05 was considered statistically significant: ^*^ *p* *<* 0.05, ^**^ *p* *<* 0.01, ^***^ *p* *<* 0.001 comparing with normoxic control, ^#^ *p* *<* 0.05, ^##^ *p* *<* 0.01, ^###^ *p* *<* 0.001 comparing with hypoxic control, ^$^ *p* *<* 0.05, ^$$^ *p* *<* 0.01, ^$$$^ *p* *<* 0.001 comparing hypoxia with normoxia, *n* ≥ 3. ^%^ *P* < 0.05, ^%%^ *P* < 0.01, ^%%%^ *P* < 0.001 comparing day 8 vs day 11.

In order to confirm that CIS-treated hypoxic lung cancer cells exhibited an active escape from senescence, time-course experiments were conducted. Cell number (**Figure 4A**) and cell cycle distribution (**Figures 4B**–**F**) on two time-points: days 8 and 11 of the experiment were analyzed. On day 8, there were no differences in cell number between normoxic and hypoxic conditions. In contrast, on day 11, the restoration of cell growth was observed in hypoxia but not in normoxia (**Figure 4A**). It was accompanied by changes in cell cycle distribution: an increase accumulation of hypoxic cells in sub-G1 (**Figure 4B**) and reduced proportion of cells in S (**Figure 4D**) and G2/M (**Figure 4E**) phases. There were no differences in cell number in other phases of cell cycle: G0/G1 phase (**Figure 4C**) or polyploidization (**Figure 4F**).

In summary, these data showed that in hypoxia, human and murine lung cancer cells escape from cisplatin-induced senescence. After initial growth inhibition, the cells escaped from the G2/M phase, proceed to the G0/G1 phase of cell cycle, and exhibited increased proliferation. It seemed to be accompanied by elevated rate of apoptotic events.

### Escape From Senescence of Hypoxic CIS-Treated Lung Cancer Cells Depended on the p53/p21 Signaling Pathway

**Figure 5:**
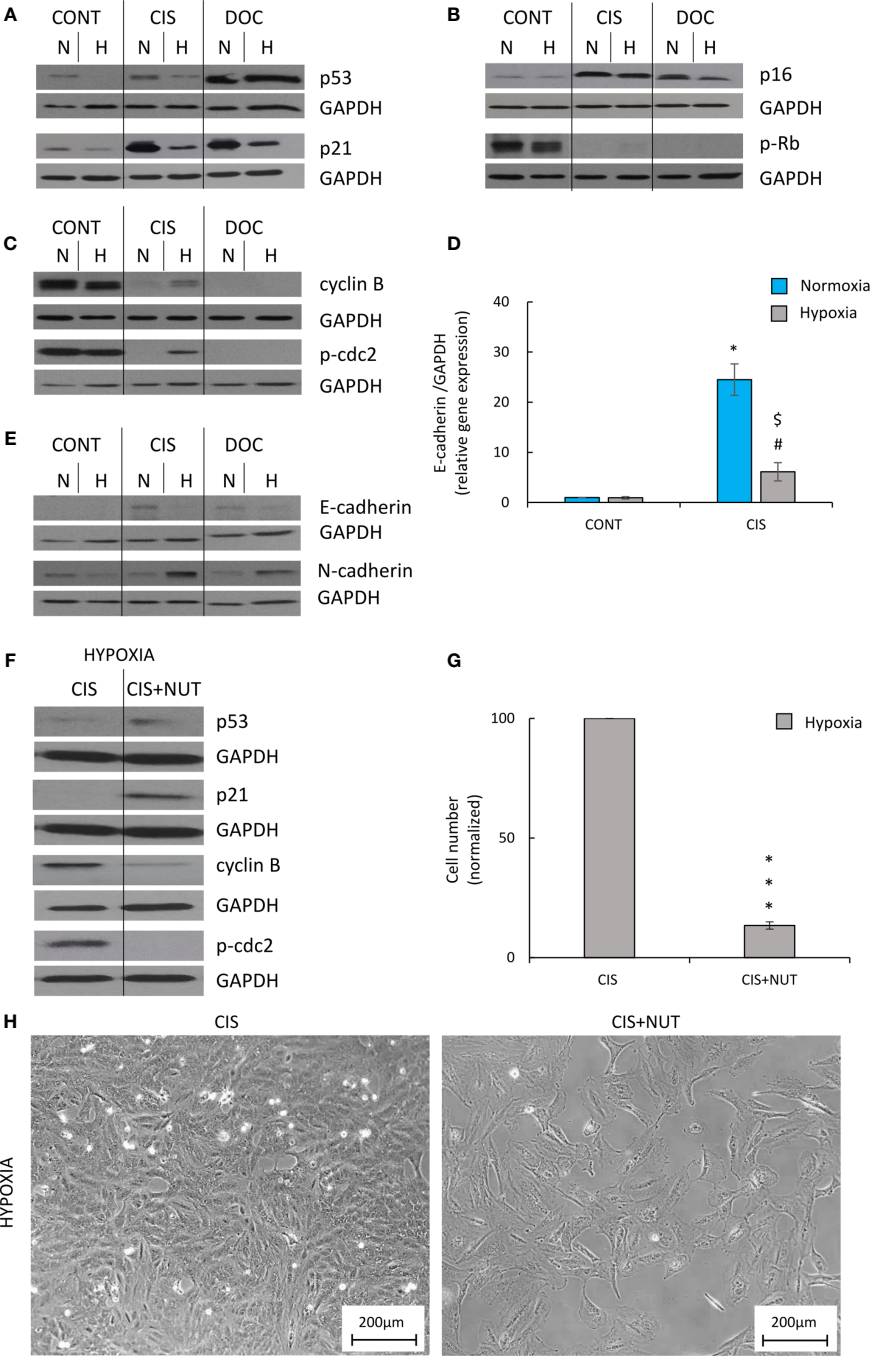
Hypoxia modulates expression of proteins related to senescence, proliferation, and EMT in CIS-treated human lung cancer, what can be inverted by p53 activation. Analysis was performed on the 11th day of the experiment (according to **Figure 1A**). Representative Western blots showing expression of p53 and p21 **(A)**, p16 and pRb **(B)**, cyclin B and p-cdc2 **(C)**, and E-cadherin and N-cadherin **(E)** after CIS or DOC treatment. GAPDH was used as a constitutive reference protein. Expression of E-cadherin gene expression was measured using qRT-PCR. GAPDH was used as a constitutive reference gene **(D)**. To test the effect of p53, lung cancer cells were treated with p53 activator, NUT together with CIS. Representative Western blots showing expression of p53, p21, cyclin B, and p-cdc2 in NUT-treated cells. GAPDH was used as a constitutive reference protein **(F)**. Evaluation of cell number after NUT treatment in hypoxia was performed using Bürker’s chamber **(G)**. Cell morphology after NUT treatment shown as representative images were acquired using light microscopy: original magnification, ×100; scale bar, 200 µm **(H)**. The respective *p*-values were calculated using a two-tailed *t*-test or Mann-Whitney *U* test, and *p* < 0.05 was considered statistically significant: ^*^ *p* *<* 0.05, ^**^ *p* *<* 0.01, ^***^ *p*-value <0.001 comparing with normoxic control, ^#^ *p* *<* 0.05, ^##^ *p* *<* 0.01, ^###^ *p* *<* 0.001 comparing with hypoxic control, ^$^ *p* *<* 0.05, ^$$^ *p* *<* 0.01, ^$$$^ *p* *<* 0.001 comparing hypoxia with normoxia, *n* ≥ 3.

To identify the potential molecular mechanism responsible for the observed differences induced by hypoxia, an analysis of protein expression, leading to senescence and proliferation, was performed. Induction of senescence depends on the activation of two major molecular pathways: p53/p21 and p16/Rb (40). CIS-treated human A549 lung cancer cells produced a significant increase in the expression of p53 (**Figure 5A**; **Supplementary Figure 2A**), p21 (**Figure 5A**; **Supplementary Figure 2B**), and p16 (**Figure 5B**; **Supplementary Figure 2C**), while expression of p-Rb was decreased (**Figure 5B**; **Supplementary Figure 2D**). Accordingly in hypoxia, the cells exhibited reduced levels of p53 (**Figure 5A**; **Supplementary Figure 2A**) and p21 (**Figure 5A**; **Supplementary Figure 2B**), suggesting that this pathway was mainly involved in escaping from CIS-induced senescence. Expression of p16 (**Figure 5B**; **Supplementary Figure 2C**) and p-Rb (**Figure 5B**; **Supplementary Figure 2D**) did not show any significant changes between the various oxygen conditions. Of importance, the levels of the two proteins that regulate progression from the G2 phase of cell cycle to mitosis–cyclin B and p-cdc2 (**Figure 5C**; **Supplementary Figure 2E**) and p-cdc2 (**Figure 5C**; **Supplementary Figure 2F**) were augmented in CIS-treated lung cancer cells under hypoxic conditions in comparison with normoxia. As we showed previously, colon cancer cells exhibited epithelial to mesenchymal transition (EMT) during escape from senescence (16, 17). Under normoxic conditions, CIS treatment of lung cancer cells also strongly elevated the expression of the epithelial marker, E-cadherin compared with untreated cells (**Figures 5D, E**; **Supplementary Figure 2G**). Subsequently, its level was decreased in hypoxia, both at the mRNA (**Figure 5D**) and protein levels (**Figure 5E**; **Supplementary Figure 2G**). Conversely, expression of the mesenchymal marker, N-cadherin showed a tendency to be upregulated in CIS-treated A549 cells under hypoxia in comparison with normoxia (**Figure 5E**; **Supplementary Figure 2H**).

DOC-treated A549 cells also exhibited an increased expression of senescence markers: p53 (**Figure 5A**; **Supplementary Figure 2A**), p21 (**Figure 5A**; **Supplementary Figure 2B**), and p16 (**Figure 5B**; **Supplementary Figure 2C**), as well as decreased expression of proliferation proteins: p-Rb (**Figure 5B**; **Supplementary Figure 2D**), cyclin B (**Figure 5C** and **Supplementary Figure 2E**), and p-cdc2 (**Figure 5C** and **Supplementary Figure 2F**). Cells cultured under hypoxic conditions showed a significant reduction in p21 expression (**Figure 5A**; **Supplementary Figure 2B**). Moreover, DOC-treated cells demonstrated an increased expression of E-cadherin in normoxia, which was downregulated in hypoxia (**Figure 5E** and **Supplementary Figure 2G**).

To verify that escaping of CIS-treated lung cancer cells from senescence is dependent on p53/p21 signaling pathway, additional treatment of senescent cells with p53 activator, NUT was applied. NUT-treated cells showed elevated expression of p53 (**Figure 5F** and **Supplementary Figure 3A**) and its downstream target, p21 (**Figure 5F**; **Supplementary Figure 3B**). It was correlated with decreased cell number (**Figure 5G**), changed cell morphology (**Figure 5H**), and reduced expression of proliferation markers: cyclin B (**Figure 5F**; **Supplementary Figure 3C**) and p-cdc2 (**Figure 5F**; **Supplementary Figure 3D**).

Taken together, these results indicated that escape from senescence in hypoxic CIS-treated lung cancer cells depended on the p53/p21 signaling pathway and the progression from the G2 phase to mitosis required activation of cyclin B. In addition, the EMT results suggested a role in escaping from senescence. Importantly, we do not specify the exact point where the senescence escape process begins: before or after senescence induction.

### Colon Cancer Cells Treated With a DNA-Damaging Agent Showed Evidence of Escape From Senescence Under Hypoxic Conditions

To verify our observations in a different cellular model, we used human colon cancer cells HCT116. As CIS is not offered to colon cancer patients in clinical practice, we tested the effects of IRINO in the same hypoxic conditions. IRINO inhibits topoisomerase I, what results in the formation of DNA breaks and the arrest of the cell cycle by activating checkpoints (41). Similar to CIS, IRINO acts on DNA and disturbs acid-base balance, which may affect the response of cancer cells to both drugs in hypoxic conditions (42).

**Figure 6:**
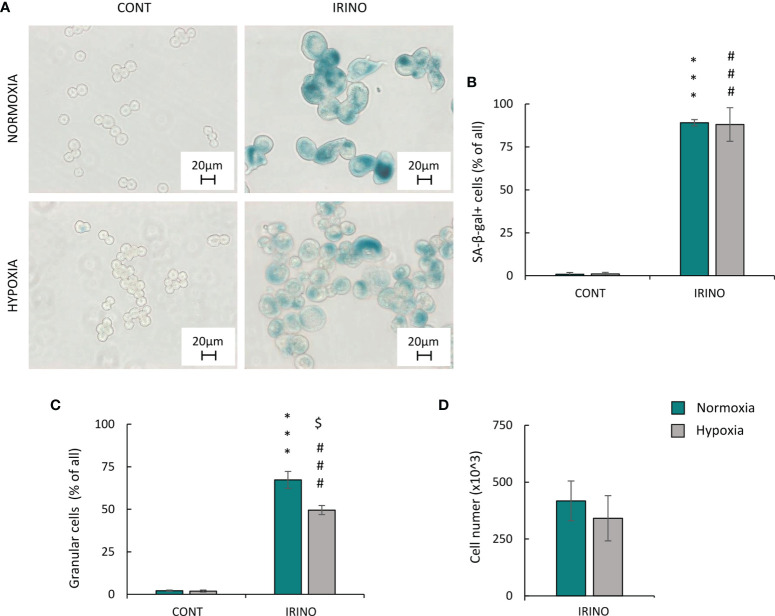
Hypoxia does not alter phenotypic markers of senescence and proliferation in IRINO-treated HCT116 colon cancer cells. Detection and quantification of senescence and proliferation markers in cells after IRINO treatment were performed on the 11th day of the experiment (according to **Figure 1A**). Detection of senescent cells after IRINO treatment was performed using SA-β-gal staining on cytospined cells. Representative photos were acquired using light microscopy: original magnification, ×400; scale bar, 20 µm **(A)**. Quantification of SA-β-gal-positive cells shown as a percentage of positive cells **(B)**. Percentages of granular cells were obtained using FSC/SSC analysis and flow cytometry **(C)**. Evaluation of cell number after chemotherapeutic drug was performed using Bürker’s chamber **(D)**. Each bar represents mean ± SEM. The respective *p*-values were calculated using two-tailed *t*-test or Mann-Whitney *U* test, and a *p*-value <0.05 was considered statistically significant: ^*^ *p* *<* 0.05, ^**^ *p* *<* 0.01, ^***^ *p* < 0.001 comparing with normoxic control, ^#^ *p* *<* 0.05, ^##^ *p* *<* 0.01, ^###^ *p* *<* 0.001 comparing with hypoxic control, *n* ≥ 3. ^$^ *P* < 0.05, ^$$^ *P* < 0.01, ^$$$^ *P* < 0.001 comparing hypoxia to normoxia.

**Figure 7:**
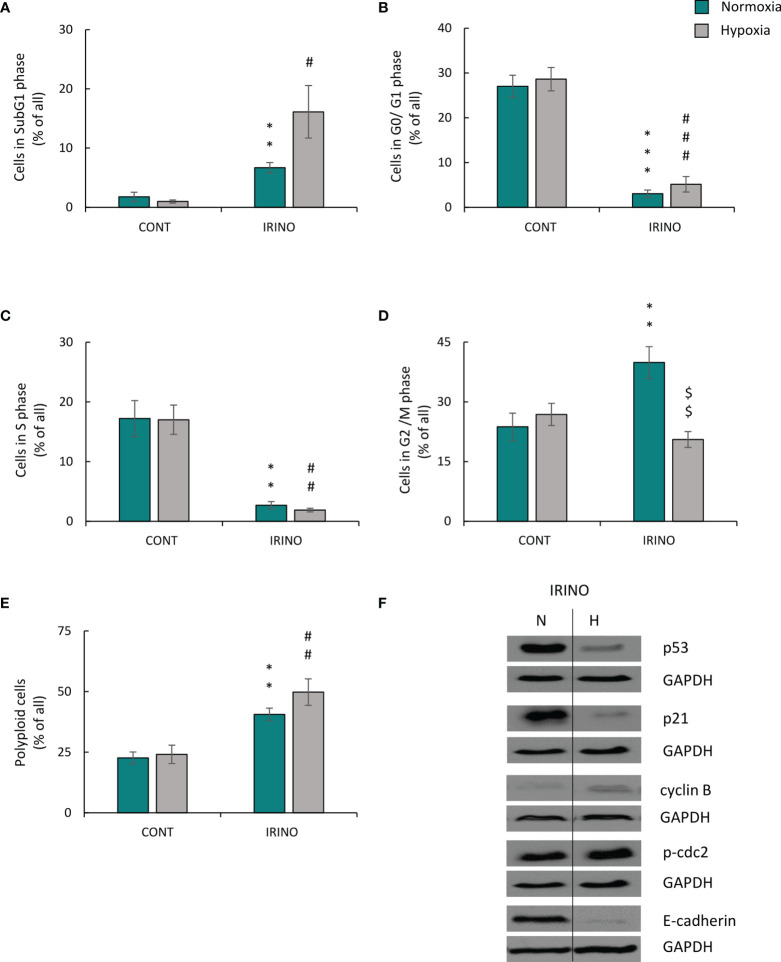
Hypoxia alters molecular markers of senescence, proliferation, and EMT in IRINO-treated colon cancer cells. Detection and quantification of senescence and proliferation markers in cells after IRINO treatment were performed on the 11th day of the experiment (according to **Figure 1A**). Cell cycle distribution after IRINO treatment was performed using PI staining and flow cytometry: sub-G1 **(A)**, G0/G1 **(B)**, S **(C)**, G2/M **(D)**, and polyploidy **(E)**. Representative Western blots showing expression of p53, p21, cyclin B, p-cdc2, and E-cadherin. GAPDH was used as a constitutive reference protein **(F)**. The respective *p*-values were calculated using two-tailed *t*-test or Mann-Whitney *U* test, and a *p*-value  <0.05 was considered statistically significant: ^*^ *p* *<* 0.05; ^**^ *p* *<* 0.01, ^***^ *p* *<* 0.001 comparing with normoxic control, ^#^ *p* *<* 0.05, ^##^ *p* *<* 0.01, ^###^ *p* *<* 0.001 comparing with hypoxic control, ^$^ *p* *<* 0.05, ^$$^ *p* *<* 0.01, ^$$$^ *p* *<* 0.001 comparing hypoxia with normoxia, *n* ≥ 3.

The cells were treated with 5 μM of IRINO for 24 h, and then they were cultured in a drug-free medium for the next 7 days. IRINO caused a significant increase in the number of SA-β-gal+ (**Figures 6A, B**) and granular (**Figure 6C**) cells. Despite the lack of significant differences in the number of cells (**Figure 6D**) and percentages of SA-β-gal-positive (**Figure 6B**) and granular (**Figure 6C**) cells between the different aerobic conditions, the appearance of the cells differed on macroscopic inspection. Namely, hypoxia reduced cell size and SA-β-gal staining intensity (**Figure 6A**). Similar to CIS-treated lung cancer cells, IRINO-treated colon cancer cells showed higher accumulation in: the subG1 (**Figure 7A**), the G2/M phase (**Figure 7D**) and polyploidization (**Figure 7E**), whereas the proportion of cells in G0/G1 (**Figure 7B**) and S phase (**Figure 7C**) was diminished. Of note, hypoxia abolished the effects of IRINO on G2/M accumulation (**Figure 7D**). Consequently, HCT116 cells cultured under hypoxic conditions showed decreased expression of the senescent markers: p53 (**Figure 7F**; **Supplementary Figure 3E**) and p21 (**Figure 7F**; **Supplementary Figure 3F**), whereas expression of proliferation proteins: cyclin B (**Figure 7F**; **Supplementary Figure 3G**) and p-cdc2 (**Figure 7F**; **Supplementary Figure 3H**) was upregulated. Moreover, hypoxia reduced expression of E-cadherin in IRINO-treated colon cancer cells (**Figure 7F**; **Supplementary Figure 3I**).

In summary, under hypoxic conditions, cancer cells treated with DNA-damaging agents showed escape from senescence, and the process seemed to be dependent on the p53/p21 pathway, progression from the G2 phase of the cell cycle to mitosis and subsequent EMT.

### Autophagy Inhibition by HCQ Reduced Escape From Senescence of CIS-Treated Lung Cancer Cells Under Hypoxic Conditions

As demonstrated previously, modulation of autophagy influences the escape from senescence in colon and glioma cancer cells both *in vitro* and *in vivo* (16). Thus, we asked the question, whether this would be observed in CIS-treated lung cancer cells cultured under hypoxic conditions.

**Figure 8:**
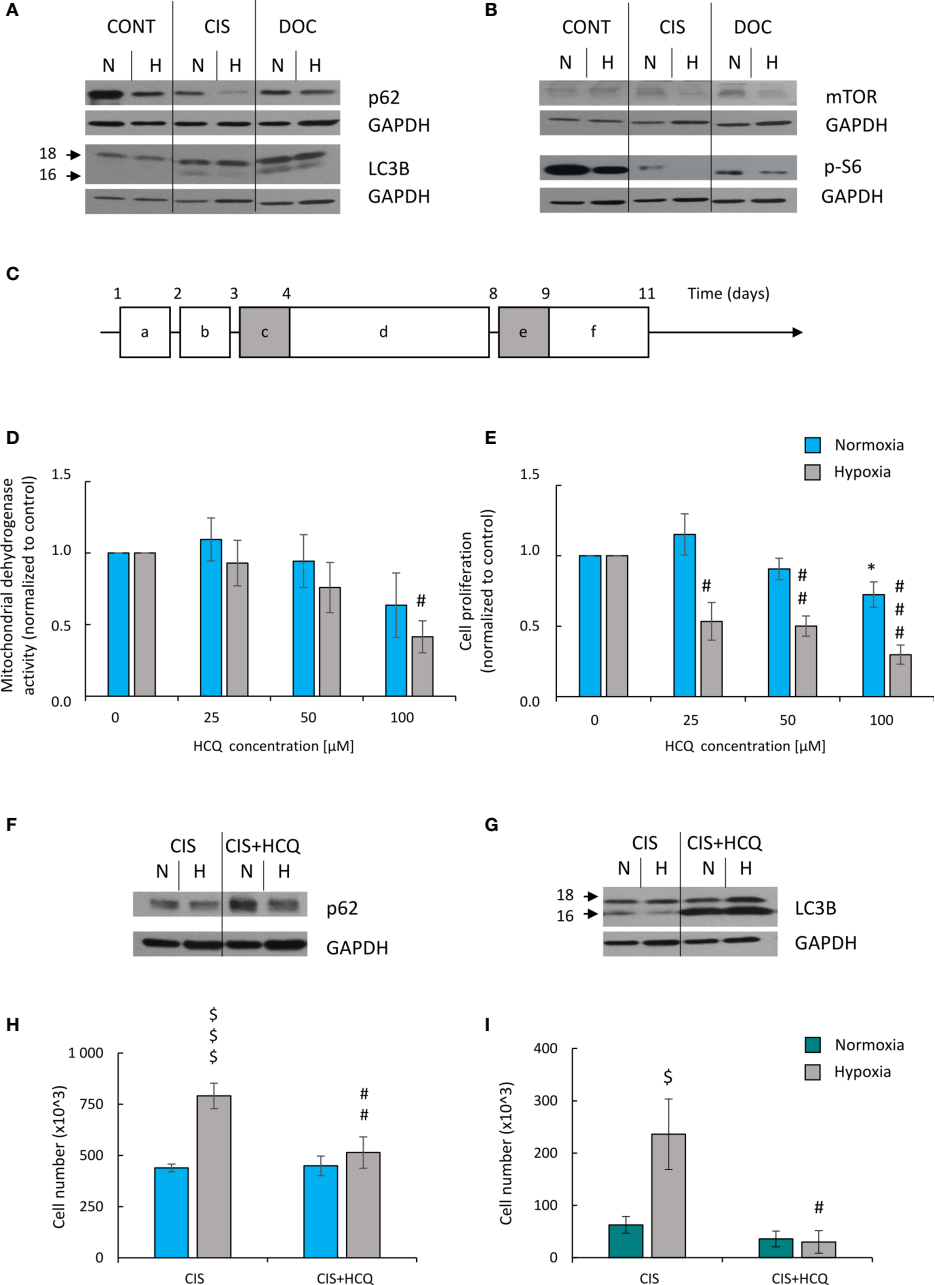
HCQ inhibits ability of hypoxic CIS-treated lung cancer cells to escape from the senescence. All analyses after CIS and HCQ treatment were performed on the 11th day of the experiment (according to scheme **C**). Representative Western blot presenting expression of p62 and LC3B II/I **(A)** and mTOR and p-S6 **(B)** proteins after CIS or DOC treatment. GAPDH was used as a constitutive reference protein. Scheme of the experiment with HCQ **(C)**. Cells were grown in normoxic condition (~19% O_2_) for 24 h (**a**). Some plates or flasks were then transferred to the hypoxic chamber, and the medium was changed (**b**). After the next 24 h, cells in normoxia or hypoxia were treated with CIS for 24 h (**c**), and then they were cultured in drug-free medium for 4 days (**d**). For the next 24 h, cells were incubated in medium with 50 µM HCQ (**e**). Medium was then changed and cells were incubated for the next 2 days (**f**). MDH test in normoxia or hypoxia **(D)** was performed to assess an effect of HCQ on cell metabolism of CIS-treated lung cancer cells. BrdU incorporation test under normoxia or hypoxia conditions **(E)** was performed to assess the effects of HCQ on cell proliferation of CIS-treated lung cancer cells. Representative Western blots showing expression of p62 **(F)** and LC3B II/I **(G)** in HCQ-treated cells. GAPDH was used as a constitutive reference protein. Cell number evaluation was performed in CIS-treated A549 **(H)** and LLC1 **(I)** cells cultured with 50 μM HCQ using Bürker’s chamber. Each bar represents mean ± SEM. The respective *p*-values was calculated using two-tailed *t*-test or Mann-Whitney *U* test, and a *p*-value  <0.05 was considered statistically significant: ^*^ *p* *<* 0.05, ^**^ *p* *<* 0.01, ^***^ *p* *<* 0.001 comparing with normoxic control, ^#^ *p* *<* 0.05, ^##^ *p* *<* 0.01, ^###^ *p* *<* 0.001 comparing with hypoxic control, ^$^ *p* *<* 0.05, ^$$^ *p* *<* 0.01, ^$$$^ *p* *<* 0.001 comparing hypoxia with normoxia, *n* ≥ 3.

Expression of two proteins related with autophagy was tested: p62/SQSTM1 (p62), which is centrally involved in the degradation of ubiquitinated cargo and LC3-binding protein (LC3B), which is directly degraded in autophagosomes during autophagy (16). Expression of p62 was reduced in untreated lung cancer cells in hypoxia (**Figure 8A**; **Supplementary Figure 4A**), suggesting that autophagy was activated. CIS treatment potentiated the effect (**Figure 8A**; **Supplementary Figure 4A**). In line, expression of another autophagic protein, LC3B II/I was increased after chemotherapeutics treatment in both oxygen conditions (**Figure 8A**; **Supplementary Figure 4B**), although CIS-treated cells showed reduced LC3B II/I ratio in hypoxia as compared with normoxia (**Figure 8A**; **Supplementary Figure 4B**). As autophagy and senescence are strongly linked to mTOR signaling pathway (43–45) expression of mTOR and its downstream target, p-S6 was checked. mTOR expression in CIS-treated cells was downregulated in hypoxic conditions compared with normoxic ones (**Figure 8B**; **Supplementary Figure 4C**). p-S6 protein showed similar pattern of expression as p62 (**Figure 8B**; **Supplementary Figure 4D**). Additionally, p-S6 expression was downregulated in DOC-treated cells both in normoxia and hypoxia (**Figure 8B**; **Supplementary Figure 4D**).

Since we observed a tendency to activate autophagy in CIS-treated lung cancer cells in hypoxia and normoxia differently (**Figures 8A, B**; **Supplementary Figures 4A**–**D**), the effects of autophagy modulation was investigated. Two approaches were applied to distinguish involvement of autophagy in induction of senescence from its effect on ability of cancer cells to escape senescence.

Effect of autophagy on induction of senescence in lung cancer cells was studied using genetic modulations. Cells were transfected with siRNAs against major autophagy-related genes: Beclin1, ATG5, or ATG7 (46) 1 day before CIS administration (**Supplementary Figure 5A**). Effectiveness of gene silencing was confirmed on the protein level (**Supplementary Figures 5B**–**E**). Knockdown of autophagy-related genes produced rather slight effects on: activity of mitochondrial dehydrogenase (**Supplementary Figure 6A**), proliferation (**Supplementary Figure 6B**), or cell number (**Supplementary Figure 6C**) both in normoxia and hypoxia. Nevertheless, the most pronounced results were observed after ATG5 and ATG7, but not Beclin1 silencing (**Supplementary Figures 6A**–**C**). There were no substantial differences in expression of p62 or LC3B II/I in cells transfected with siRNAs against autophagy-related genes (**Supplementary Figures 6D**–**F**), which may be due to a compensatory effect by other autophagy-related genes.

**Figure 9:**
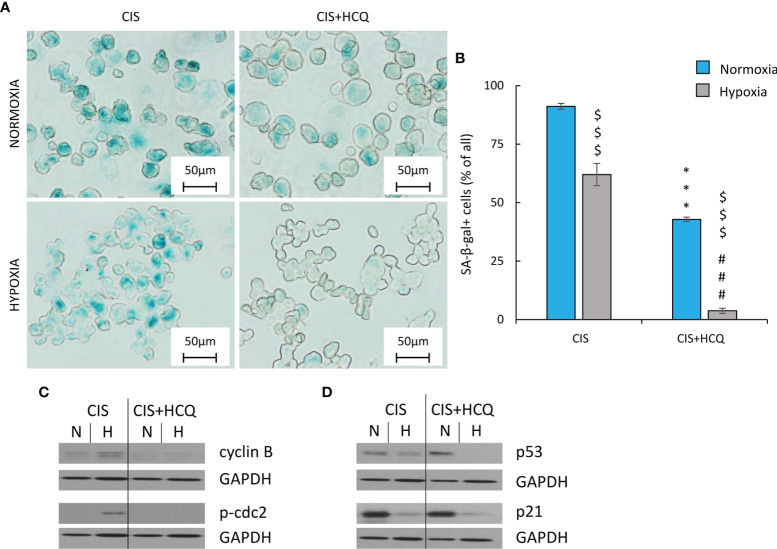
HCQ decreases expression of proliferation but not senescence markers in hypoxic CIS-treated lung cancer cells. All analyses after HCQ and CIS treatment were performed on the 11th day of the experiment (according to **Figure 8C**). Evaluation of senescent cells was conducted using SA-β-gal staining on cytospined A549 cells. Representative photos were acquired using light microscopy: original magnification, ×400; scale bar, 50 µm **(A)**. Quantification of SA-β-gal-positive cells shown as a percentage of positive cells **(B)**. Representative Western blot showing expression of cyclin B and p-cdc2 **(C)** and p53 and p21 **(D)** after HCQ treatment. GAPDH was used as a constitutive reference protein. Each bar represents mean ± SEM. The respective *p-*values was calculated using two-tailed *t*-test or Mann-Whitney *U* test, and a *p*-value  <0.05 was considered statistically significant: ^*^ *p* *<* 0.05, ^**^ *p* *<* 0.01, ^***^ *p* *<* 0.001 comparing with normoxic control, ^#^ *p* *<* 0.05, ^##^ *p* *<* 0.01, ^###^ *p* *<* 0.001 comparing with hypoxic control, ^$^ *p* *<* 0.05, ^$$^ *p* *<* 0.01, ^$$$^ *p* *<* 0.001 comparing hypoxia with normoxia, *n* ≥ 3.

Next, effect of autophagy modulation on ability of cancer cells to escape senescence was investigated. As senescent cells are hardly transfected nondividing cells, the pharmacological inhibitor HCQ was applied. HCQ blocks the late phase of autophagy, as it prevents the maturation of autophagic vacuoles by inhibiting the fusion between autophagosomes and lysosomes (47). To test, whether autophagy takes part in escaping from senescence, CIS-treated cells were cultured in the presence of 50 µM HCQ for 24 h. Next, the inhibitor was removed and cells were cultured in drug-free medium for the next 3 days (**Figure 8C**). In response to HCQ, CIS-treated lung cancer cells reduced mitochondrial dehydrogenase (**Figure 8D**) and proliferative activity (**Figure 8E**) but under hypoxic conditions only (**Figures 8D, E**). Protein expression analysis showed that treatment with HCQ perturbed the autophagic flux, shown as p62 accumulation (**Figure 8F**; **Supplementary Figure 7A**) and LC3B activation (**Figure 8G**; **Supplementary Figure 7B**). Although, hypoxia seemed to reduce expression of p62 in HCQ-treated senescent cells (**Figure 8F**; **Supplementary Figure 7A**). Inhibition of autophagy by HCQ was translated into a decrease in the number of proliferating cells in hypoxia both in human (**Figure 8H**) and murine (**Figure 8I**) lung cancer cell lines. When investigating the activity of SA-β-gal, the cells stained weakly after HCQ treatment, but the characteristic features of TIS cells were retained [**Figures 9A, B**, discussed already in (16)]. Accordingly, the expression of proliferative proteins: cyclin B (**Figure 9C**; **Supplementary Figure 7C**) and p-cdc2 (**Figure 9C**; **Supplementary Figure 7D**), despite their induction by hypoxia, significantly decreased upon HCQ actions. No significant difference was observed for senescence pathway-associated proteins: p21 (**Figure 9D**; **Supplementary Figure 7E**) or p53 (**Figure 9D**; **Supplementary Figure 7F**). HCQ was applied also to nonsenescent lung cancer cells. HCQ produced some toxic effect to nonsenescent cells under hypoxia, presented as increased proportion of cells in sub-G1 phase (**Supplementary Figure 7G**) and reduced cell number (**Supplementary Figure 7H**).

**Figure 10:**
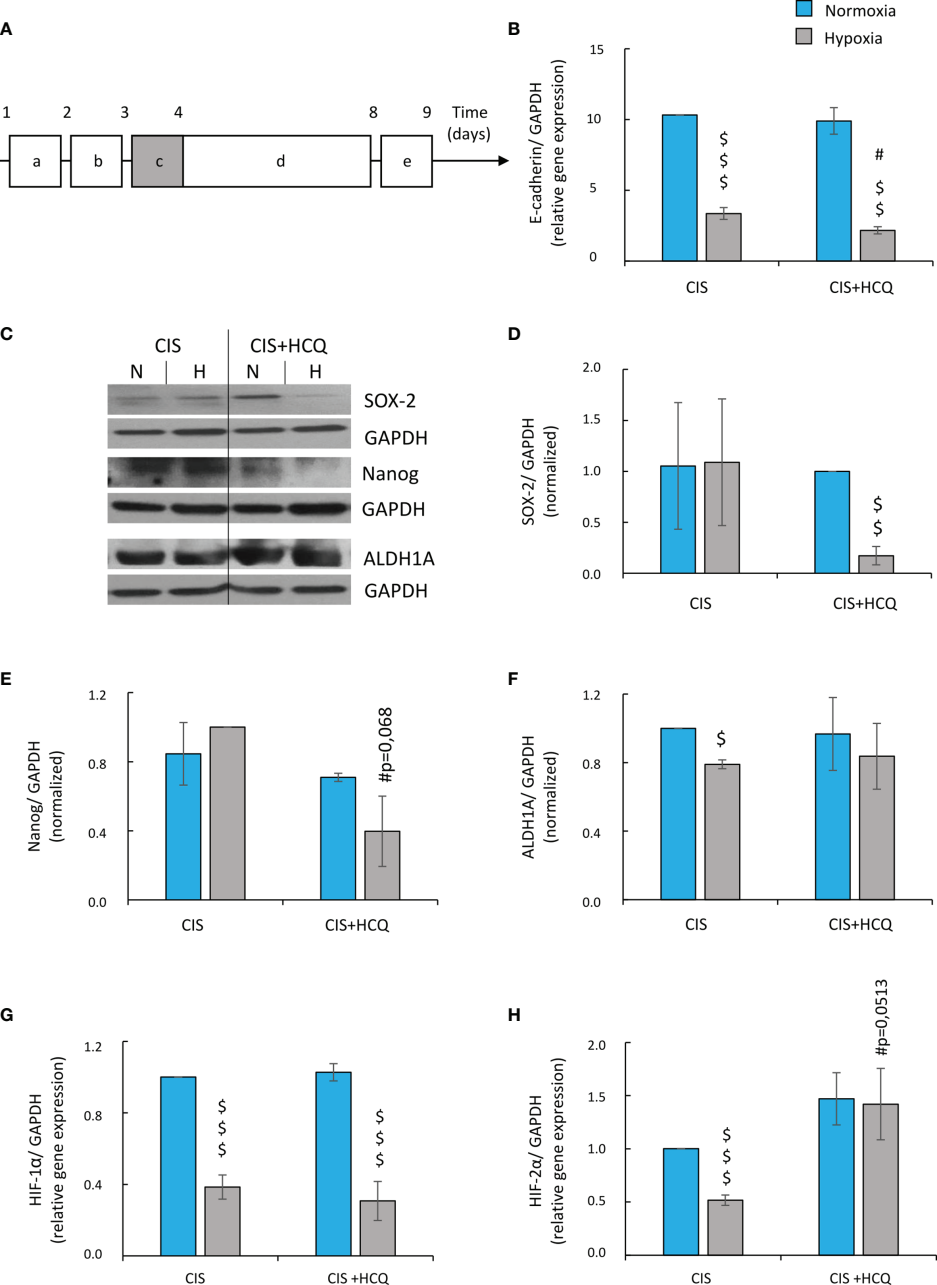
HCQ reduces expression of E-cadherin and changes expression of hypoxia and stemness-related genes in CIS-treated lung cancer cells under hypoxic conditions. All analyses after HCQ and CIS treatment were performed on the 9th day of the experiment (according to scheme). Scheme of the experiment **(A)** (10A). Cells were grown in normoxic condition (~19% O_2_) for 24 h **(a)**. Some plates or flasks were then transferred to the hypoxic chamber, and the medium was changed **(b)**. After the next 24 h, cells in normoxia or hypoxia were treated with CIS for 24 h **(c)** and then they were cultured in drug-free medium for 4 days **(d)**. For the next 24 h, cells were incubated in medium with HCQ at 50 µM concentration **(e)**. Expression of E-cadherin gene was measured using qRT-PCR. GAPDH was used as a reference gene **(B)**. Representative Western blots showing expression of SOX-2, Nanog, and ALDH1A in HCQ-treated cells **(C)**. Quantification of Western blotting results was performed using densitometric analysis and ImageJ software. Data shown as a ratio of the respective protein: SOX-2 **(D)**, Nanog **(E)** ALDH1 **(F)** to a loading control (GAPDH). Expression of HIF-1α **(G)** and HIF-2α **(H)** genes was measured using qRT-PCR. GAPDH or PPIA was used as a reference gene. The respective *p*-values was calculated using two-tailed *t*-test or Mann-Whitney *U* test, and a *p*-value  <0.05 was considered statistically significant. *p *<* 0.05, ^**^ *p* *<* 0.01, ^***^ *p* *<* 0.001 comparing with normoxic control, ^#^ *p* *<* 0.05, ^##^ *p* *<* 0.01, ^###^ *p* *<* 0.001 comparing with hypoxic control, ^$^ *p* *<* 0.05, ^$$^ *p* *<* 0.01, ^$$$^ *p* *<* 0.001 comparing hypoxia with normoxia, *n* ≥ 3.

Twenty-four hours after HCQ treatment (**Figure 10A**), the expression of E-cadherin mRNA in CIS-treated A549 cells was further reduced under hypoxic conditions (**Figure 10B**), suggesting acquisition of mesenchymal phenotype (EMT). As this phenotype is one of a characteristic of cancer stem cells (CSCs) (48, 49), stemness-related markers were tested: OCT-3/4, NANOG, SOX2, and ALDH1. HCQ reduced expression of SOX-2 and NANOG in hypoxic CIS-treated cells both on mRNA (**Supplementary Figures 8A, B**) and protein level (**Figures 10C**–**E**). ALDH1 mRNA was also downregulated in senescent cancer cells after HCQ treatment (**Supplementary Figure 8C**), but expression of its protein was not changed significantly (**Figure 10F**). Finally, HCQ did not affect expression of OCT-3/4 in hypoxic cells (**Supplementary Figure 8D**). As both, EMT process and appearance of CSCs might be related with hypoxia (50–55), expression of hypoxia-inducible factors, HIF-1α and HIF-2α were analyzed. HCQ elevated the expression of HIF-2α (**Figure 10H**) in CIS-treated cells but not of the HIF-1α gene (**Figure 10G**).

Taken together, HCQ reduced the escape from senescence of CIS-treated lung cancer cells under hypoxia. Moreover, exposure to HCQ led to EMT and, when associated with hypoxia, it modulated distinctly combinations of cancer stem cell markers.

## Discussion

Enhanced proliferation and elevated metabolism of cancer cells as well as inefficient angiogenesis may lead to hypoxia within tumor tissues (56). This phenomenon plays a significant role in cancer chemoresistance and is partly responsible for therapy failure (57). Hypoxia takes part in the chemoresistance of different types of cancers to DNA-damaging agents, including cisplatin (58–63). Cisplatin forms different adducts with DNA (monoadducts, intrastrand crosslinks, DNA-protein crosslinks) activating DDR, cell cycle arrest, and apoptosis. Additionally, CIS induces mitochondrial damage, resulting in excessive reactive oxygen species (ROS) generation and lipid peroxidation. Finally, cisplatin toxicity involves disruption of calcium signaling, disruption of the Na+/H+ membrane pump, and inhibition of the Na+/K+ ATPase pump (64). All these mechanisms can interfere with chemoresistance of cancer cells under hypoxic conditions. However, there are very few reports linking hypoxia with cellular senescence (65). Therefore, herein, we investigated the effects of hypoxia on the therapy-induced senescence of lung cancer cells, considering its potential role in the control of the deleterious escape from senescence, which is linked to cells being subjected to autophagy modulation. Our findings showed that: (i) lung cancer cell cultures exhibited higher resistance to cisplatin in hypoxic than in normoxic conditions; (ii) upon exposure to hypoxic conditions, lung cancer cells escaped from cisplatin-induced senescence, which relied on the p53/p21 signaling pathway; and (iii) autophagy inhibition by HCQ reduced escape from senescence of CIS-treated lung cancer cells in hypoxia.

We observed that human (A549) and murine (LLC1) lung cancer cell lines exhibited higher resistance to cisplatin in hypoxic compared with normoxic conditions. Moreover, cisplatin treatment led to the induction of senescence, which was associated with mesenchymal to epithelial transition (MET). Furthermore, hypoxia reversed this phenotype, leading to escape from senescence. The question remains open: at what senescence stage does this escape occurs. Mechanistically, resistance was accompanied by alterations in the major senescence pathway, p53/p21, as confirmed by experiments with nutlin-3, p53 activator. Accordingly, Cramer and coworkers concluded that activation of HIF-1α in cisplatin-treated gastric cancer led to reduced cellular senescence and apoptosis by suppressing p53 and p21. Silencing HIF-1α with RNA interference increased cancer cell chemosensitivity (65). Additionally, mesenchymal stromal cells (MSCs) showed an increase in the resistance to genotoxic damage under hypoxic conditions. This was accompanied by a decrease in the expression of p53 and p21 and reduced accumulation of cells in the G2/M phase. Furthermore, MSCs with shRNA-mediated p53 knockdown exhibited downregulated cell cycle arrest and increased cyclin B/cdc-2 expression. However, p53 knockdown did not modulate resistance to cisplatin (66). A preoxygenated perfluorooctyl bromide (PFOB) nanoemulsion with carbogen breathing effectively increased tumor oxygenation in mouse model, which associated with an increase in cisplatin-mediated apoptosis of cancer cells and inhibition of tumor growth with low doses of cisplatin treatment (67).

We verified our observations relative to hypoxia-induced escape from senescence using another experimental model. As cisplatin is not used for treatment of colon cancer, human HCT116 colon cancer cells were treated with IRINO, which is a different DNA-damaging agent. This camptothecin derivative targets topoisomerase-1 and is largely used in combination regimens to treat metastatic colon cancer (68). Interestingly, we did not observe any significant phenotypic changes across oxygen conditions. Although, hypoxic IRINO-treated colon cancer cells displayed altered expression of senescence, proliferation, and EMT markers, which suggested an early stage of escape from senescence. Accordingly, Chintala et al. indicated that the degradation of HIF-1α sensitized cancer to IRINO treatment in animal model (69). Additionally, resistance to IRINO was correlated with lack of microvessel formation, hypoxia, and limited drug delivery in mice bearing head and neck squamous cell carcinoma (HNSCC) xenografts (70). We and others showed that colon cancer cells underwent senescence in response to IRINO and could escape, which contributed to cancer repopulation (17, 71). Interestingly, Yes-Associated Protein (YAP), a key mediator of the Hippo pathway, has been proposed as a novel mediator of hypoxic resistance to SN38 (active metabolite of IRINO) in hepatocellular carcinoma. The induction of YAP by hypoxia was not mediated by HIF-1α, but rather by the mevalonate-HMG-CoA reductase (HMGCR) pathway (72). Moreover, the embryonic-like stemness characteristic of polyploid giant cells (PGCCs) was associated with nuclear accumulation of YAP in ovarian cancer cells (24). Accordingly, we previously observed that rare, polyploid colon cancer cells might develop a blastocyst-like morphology and produce progeny (17). A critical feature of PGCCs is the generation of progeny during depolyploidization, which may display marked aggressiveness, resulting in the formation of resistant disease and tumor recurrence (19).

Subsequently, we compared data obtained from cells treated with the DNA-damaging agents, cisplatin and IRINO to data obtained from cells treated with DOC, which has a different mode of action. DOC is a second-generation taxane that promotes microtubulin assembly and stabilizes polymers against depolymerization. In turn, this leads to a significant decline in free tubulin, needed for microtubule formation and causes inhibition of mitotic cell division between metaphase and anaphase (73, 74). We showed that DOC induced senescence of A549 lung cancer cells both in normoxia and hypoxia, but there were no significant differences in resistance to the drug in either of these oxygen conditions. Although, we did not observe any phenotypic changes related to escape from senescence, a reduction in the expression of p21 and E-cadherin proteins was observed upon exposure to hypoxia. It might suggest a very early stage of escape from senescence. In accordance, DOC caused senescence of cancer cells and the inhibition of this process affected the resistance to the drug under normoxic conditions (75, 76). Moreover, HIF-1α increased resistance to taxanes (77–79). However, there is no clear evidence linking this effect with TIS. Additionally, propofol (2,6-diisopropylphenol) reversed the hypoxia-induced DOC resistance in prostate cancer. Propofol reduced hypoxia-induced HIF-1α expression and reversed hypoxia-induced EMT by inhibiting HIF-1α (80). Interestingly, Peng et al. showed that normoxic breast cancer cells utilized a crosstalk between EGFR signals and HIF-1α to increase the expression of the antiapoptotic gene, survivin. Targeting HIF-1α could be considered a therapeutic approach for both hypoxic and normoxic tumor cells (81). In turn, survivin was demonstrated to cooperate with cdc-2/cdk1 to inhibit apoptosis following chemotherapy and promote senescence escape (82). We achieved the senescence effect for DOC at much lower doses (3 nM) compared with CIS (8 µM) and IRINO (5 µM) and the cells after DOC showed no clear phenotypic signs of recovery from senescence throughout the experiment. Therefore, we hypothesize that DOC is a stronger inducer of senescence than CIS or IRINO and inhibit escaping from senescence more efficiently than DNA-damaging agents. It might be related with its mode of action. DOC controls processes that are crucial for cell division, namely: assembly of mitotic spindle and separation of the sister chromatids of chromosome. These could be critical also for cell polypliodization/depolyploidization and formation of progeny by senescent cancer cells. Moreover, our data suggest that docetaxel mode of action is not as much dependent of oxygen tension as cisplatin or irinotecan. Nevertheless, our observations and hypotheses require further studies and confirmation.

As we demonstrated previously, modulation of autophagy affects the escape from senescence in colon cancer cells both *in vitro* and *in vivo* (16). Inhibiting autophagy to overcome resistance to chemotherapy has been investigated in clinical trials, where HCQ has been combined with different chemotherapeutics (83–85). The relationship between autophagy and chemoresistance under hypoxic conditions has been identified in several types of cancer (86–88), including resistance to cisplatin (89, 90). Accordingly, hypoxia significantly protected A549 and SPC-A1 lung cancer cells from cisplatin-induced cell death in a HIF-1α- and HIF-2α-dependent manner. When autophagy was inhibited by 3-methyladenine (3-MA) or siRNA targeting ATG5, this reduction was effectively diminished (86). Additionally, exposure of A549 cells to hypoxia stimulated autophagic induction and resistance of cancer cells to cisplatin, and LC3B siRNA restored the sensitivity of cancer cells to chemotherapy. Of note, human lung cancer tissues that experienced chemotherapy exhibited an increase rate of autophagy compared with chemo-naive cancer (88). Though, the effects of TIS on acquired drug resistance and the role of inhibition of autophagy on cellular senescence under hypoxic conditions have not been studied in detail. Therefore, here, we focused on effect of autophagy inhibition on escaping of cancer cells from senescence in hypoxia. In a recent study by Saleh and coworkers, neither shRNAs against ATG5 nor BAF A1/HCQ treatment prior to the subsequent exposure to chemotherapeutics or radiation affected ability of cancer cells to induce senescence or recover from it (91). In line, here, we showed that siRNAs against: ATG5, ATG7, or Beclin1 introduced to normoxic or hypoxic lung cancer cells prior to CIS treatment, did not changed their ability to induce senescence or senescence escaping. Therefore, we investigated the effect of autophagy inhibition on elimination of already senescent cells and/or their ability to reactivate proliferation. Wakita et al. showed that upregulation of autophagy removed senescent hepatic stellate cells in obese mouse livers (92). Here, we demonstrated that HCQ could prevent hypoxic CIS-induced lung cancer cells from senescence escaping. HCQ significantly reduced the number of cells and the expression of proliferation markers but did not change the expression of senescence markers. In the same time, HCQ toxicity to nonsenescent lung cancer cells was slight. In contrast, our previous studies on colon and glioma cancer cells indicated that senescent cells were more resistant to BAF A1 than nonsenescent ones (16). Therefore, the question remains, whether the globally measured inhibition of cells escaping from TIS, when treated with autophagy inhibitors, is a result of the following: (i) elimination of senescent cancer cells, (ii) elimination of nonsenescent proliferating cancer cells (maternal cells, progeny), (iii) reduced ability of senescent cancer cells to re-activate proliferation and produce progeny, or (iiii) any combinations of above.

Twenty-four hours after HCQ administration to CIS-treated lung cancer cells, we observed induction of HIF-2α, but not HIF-1α gene expression. Expression of HIF is time dependent, peaks after 24 h in hypoxia. HIF-1α is the earlier and HIF-2α later factor induced by hypoxia (86, 93). Our long-term experimental conditions were likely the reason why we did not observe any upregulation of HIFs. However, since cells were treated with HCQ 1 day before being harvested, its effects on the expression of HIFs could still be detected. Accordingly, Saint-Martin et al. demonstrated that synchronized autophagy and mTOR inhibition could increase cell death and tumor remission was activated only in HIF-2α-silenced cells. Nonetheless, they showed that the HIF-2α inhibitor alone or in combination with drugs in patient-derived primary colon cancer cells was able to overcome resistance to 5-fluorouracil (5-FU) or mTOR inhibitor, CCI-779 (94). Therefore, HCQ *via* HIF-2α upregulation can lead to the development of secondary resistance. Accordingly, we showed that expression of the epithelial marker, E-cadherin in CIS-treated hypoxic A549 cells was further reduced upon HCQ treatment, suggesting appearance of cells with mesenchymal phenotype. The mesenchymal phenotype is one of the characteristics of cancer stem cells mediating the increased metastatic potential and chemoresistance of cancer cells (95, 96). Thus, we tested the stemness markers: OCT-3/4, NANOG, SOX2, and ALDH1, which showed a mixed pattern of modulation. Accordingly, our previous study showed that colon cancer cells treated with another autophagy inhibitor BAF A1 postponed the *in vitro* cell repopulation in the short term. Although, with long-term treatment, a pulse of BAF A1 induced the reactivation of autophagy and proliferation, which was correlated only with a transient decrease in the expression of cancer stem cell markers. Notably, senescent HCT116 cells treated with BAF A1 injected into NOD/SCID mice, formed tumors faster than their untreated counterparts (16). Consequently, it was demonstrated that the autophagy/lysosomal pathway is involved in chromatin processing in senescent cells (97) and in the segregation of functional subnuclei during their amitotic divisions (98). Finally, Rahim et al. showed that glioblastoma cells activated ATG9A-related autophagy as a survival mechanism to the hypoxic environment and HCQ treatment *in vivo* significantly increased survival of patient-derived xenotransplants (99). Altogether, we believe that long-term consequences of chemotherapeutics combined with autophagy modulators should by tested in the context of TIS and stemness induction.

## Conclusions

We showed that hypoxia increased the resistance of lung and colon cancer cell lines to the DNA-damaging agents, CIS and IRINO and increased the escape from senescence. In contrast, hypoxia had no direct effect on the microtubule modulator, DOC. Hypoxia and senescence are thus crucial in the modulation of chemoresistance. This study also considered the cancer cell type, drug concentration, and treatment duration. Thus, we propose these parameters are key points to consider for a personalized medicine approach.

Inhibition of autophagy by HCQ treatment may be used to overcome CIS-induced resistance in hypoxia in a time-dependent manner. Nevertheless, HCQ treatment may lead to development of secondary resistance. This opens new study possibilities for the long-term consequences of such combined treatment in preclinical and clinical trials.

We propose that escape from senescence of epithelial cancers is a multistage process, which can be followed during its development by a pattern of specific markers as summarized (**Table 1**). The early stage of escape from senescence is associated with a decrease in the expression of the cell cycle inhibitor, p21 and the EMT protein, E-cadherin. There is then a decrease in expression of another cell cycle inhibitor, p53, followed by the upregulation of cyclin B and p-cdc2, which are responsible for transition from G2 to M phases. Finally, cells proceed to the G0/G1 phase and phenotypic changes (a rise in cell number, reduced granularity and SA-β-gal activity) are evident. Although, our proposed mechanism of escape from senescence requires further verification using other experimental models. Additionally, it may be helpful to determine whether the reason for senescence escaping is its weaker induction or whether it is based on later events. We believe this algorithm to be useful in preclinical and clinical studies as a prognostic and diagnostic tool to improve the effectiveness of anticancer treatment and to decrease undesired adverse effects.

**Table 1:** Proposed molecular and cellular algorithm for evaluation the stages of escaping from TIS.

Marker	Senescence-escaping stages		
	I	II	III
↑ Cell number	−	−	+
↓ SA-β-gal	−	−	+
↓ Granularity	−	−	+
↓ Hypertrophy	−	NT	+
↑ G0/G1	−	−	+
↓ G2/M	−	+	+
↓ p53	−	+	+
↓ p21	+	+	+
↑ Cyclin B	−	+	+
↑ p-cdc2	−	+	+
↓ E-cadherin	+	+	+

NT, Not Tested.

## Data Availability Statement

The original contributions presented in the study are included in the article/**Supplementary Material**. Further inquiries can be directed to the corresponding author.

## Author Contributions

Conceptualization: HW, AO, and CK. Investigation: AO, AB, MG, JK, and BZ. Data analysis: AO, HW, CK, MG, and JK. Funding acquisition: HW and CK. Project administration: HW. Supervision: HW and CK. Writing—original draft: HW and AO. Writing—review and editing: CK, AB, MG, JK, and BZ. All authors read and accepted the final version of the manuscript.

## Funding

Research was supported by grant Sonata Bis 7 no. 2017/26/E/NZ3/00434 from National Science Centre, Poland (HW) and intramural grant no. 1/8971(500) from Military Institute of Medicine (HW). AB is the recipient of a scholarship from Postgraduate School of Molecular Medicine funded by European Union resources under the European Social Fund - Operational Program Knowledge, Education and Development under the project “Program of Interdisciplinary Doctoral Studies using new-generation sequencing (NGS) in personalized medicine.” M.G. is partially supported by Doctoral School of Translational Medicine, Centre of Postgraduate Medical Education, Warsaw, Poland.

## Conflict of Interest

The authors declare that the research was conducted in the absence of any commercial or financial relationships that could be construed as a potential conflict of interest.

## Publisher’s Note

All claims expressed in this article are solely those of the authors and do not necessarily represent those of their affiliated organizations, or those of the publisher, the editors and the reviewers. Any product that may be evaluated in this article, or claim that may be made by its manufacturer, is not guaranteed or endorsed by the publisher.

## Supplementary Material

Supplementary Figure 1 Hypoxia reduces markers of senescence and increases proliferation of CIS-treated murine LLC1 lung cancer cells. All analyses of CIS-treated murine LLC1 lung cancer cells were performed on 11^th^ day of the experiment (according to **Figure 1A**). MDH test in normoxia **(A)** was performed for assessment of CIS effect on cell metabolism. BrdU incorporation test in normoxia **(B)** was performed to assess CIS effects on cell proliferation. Detection and quantification of senescent cells using SA-β-gal staining was performed on cytospined LLC1 cells. Representative photos for cells were acquired using light microscopy: original magnification - 400x, scale bar - 20 µm **(C)**. Quantification of SA-β-gal positive cells was showed as a percentage of positive cells **(D)**. Cell numbers were determined using a Bürker’s chamber **(E)**. Each bar represents mean ± SEM. The respective *P-*values were calculated using two-tailed t-test or Mann-Whitney test and a *P*-value  < 0.05 was considered statistically significant. **P <* 0.05, ***P <* 0.01, ****P <* 0.001 comparing to normoxic control, #*P <* 0.05, ##*P <* 0.01, ###*P <* 0.001 comparing to hypoxic control, $*P <* 0.05, $$*P <* 0.01, $$$*P <* 0.001 comparing hypoxia to normoxia, n ≥ 3.

Supplementary Figure 2 Evaluation of protein expression related with senescence, proliferation and EMT in CIS- or DOC-treated A549 lung cancer cells. All analyses after CIS treatment were performed on 11^th^ day of the experiment (according to **Figure 1A**). Quantification of western blotting results was performed using densitometric analysis and ImageJ software. Data shown as a ratio of the respective protein to a loading control (GAPDH): p53 **(A)**, p21 **(B)**, p16 **(C)**, pRb **(D)**, cyclin B **(E)**, p-cdc2 **(F)**, E-cadherin **(G)** and N-cadherin **(H)**. Each bar represents mean ± SEM. The respective *P-*values were calculated using two-tailed t-test or Mann-Whitney test and a *P*-value  < 0.05 was considered statistically significant. **P <* 0.05, ***P <* 0.01, ****P <* 0.001 comparing to normoxic control, #*P <* 0.05, ##*P <* 0.01, ###*P <* 0.001 comparing to hypoxic control, $*p* value < 0.05, $$*P <* 0.01, $$$*P <* 0.001 comparing hypoxia to normoxia, n ≥ 3.

Supplementary Figure 3 Evaluation of expression of proteins related with senescence, proliferation and EMT in NUT- and CIS-treated A549 lung cancer or IRINO-treated HCT116 colon cancer cells. All analyses were performed on 11^th^ day of the experiment (according to **Figure 1A**). Quantification of western blotting results was performed using densitometric analysis and ImageJ software. Data shown as a ratio of the respective protein to a loading control (GAPDH). In NUT+CIS experiments: p53 **(A)**, p21 **(B)**, cyclin B **(C)**, p-cdc2 **(D)**, E-cadherin **(E)** and in IRINO experiments: p53 **(E)**, p21 **(F)**, cyclin B **(G)**, p-cdc2 **(H)** and E-cadherin **(I)**. Each bar represents mean ± SEM. The respective *P-*values were calculated using two-tailed t-test or Mann-Whitney test and a *P*-value  < 0.05 was considered statistically significant. $*P <* 0.05, $$*P <* 0.01, $$$*p* value < 0.001 comparing hypoxia to normoxia, n ≥ 3.

Supplementary Figure 4 Evaluation of expression of proteins associated with autophagy as well as mTOR signaling pathway in CIS- and DOC-treated A549 lung cancer cells. All analyses were performed on 11^th^ day of the experiment (according to **Figure 1A**). Quantification of western blotting results was performed using densitometric analysis and ImageJ software. Data shown as a ratio of the respective protein: p62 **(A)**, LC3B II/I **(B)**, mTOR **(C)** and p-S6 **(D)** to a loading control (GAPDH). Each bar represents mean ± SEM. The respective *P-*values was calculated using two-tailed t-test or Mann-Whitney test and a *P*-value  < 0.05 was considered statistically significant. **P <* 0.05, ***P <* 0.01, ****p* value < 0.001, #*P <* 0.05, ##*P <* 0.01, ###*P <* 0.001 comparing to hypoxic control, $*P* < 0.05, $$*P <* 0.01, $$$*P <* 0.001 comparing hypoxia to normoxia, n ≥ 3.

Supplementary Figure 5 Evaluation of efficacy of silencing autophagy-related genes with siRNAs in CIS-treated lung cancer cells. All analyses were performed on 12^th^ day of the experiment (according to **A**). Scheme of the experiment (5A). Cells were grown in normoxic condition (~19% O_2_) for 24 hours **(a)**. Then, some plates or flasks were transferred to the hypoxic chamber and the medium was changed **(b)**. Then, autophagy-related gene encoding: BCN1, ATG5 and ATG7 were silenced using siRNAs **(c)**. After next 24 hours the cells were treated with CIS for 24 hours **(d)** and subsequently cultured in drug-free medium for 4 days **(e)**. Next, medium was changed and cells were incubated for 3 days **(f)**. Western blots with GAPDH as a loading control were performed to confirm ATG5, ATG7 or BCN gene silencing **(B)**. Quantification of western blotting results was performed using densitometric analysis and ImageJ software. Data shown as a ratio of a respective protein: ATG5 **(C)**, ATG7 **(D)** or BCN1 **(E)** to a loading control (GAPDH). Each bar represents mean ± SEM. The respective *P-*values was calculated using two-tailed t-test or Mann-Whitney test and a *P*-value  < 0.05 was considered statistically significant. **P <* 0.05, ***P <* 0.01, ****p* value < 0.001, #*P <* 0.05, ##*P <* 0.01, ###*P <* 0.001 comparing to hypoxic control, $*P* < 0.05, $$*P <* 0.01, $$$*P <* 0.001 comparing hypoxia to normoxia, n ≥ 3.

Supplementary Figure 6 Effect of BCN1-, ATG5- and ATG7-silencing on metabolism, proliferation and autophagy of CIS-treated lung cancer cells. All analyses were performed on 12^th^ day of the experiment (according to **Supplementary Figure 5A**). MDH test in normoxia or hypoxia **(A)** was performed to assess an effect of gene silencing on the cell metabolism of CIS-treated lung cancer cells. BrdU incorporation test in normoxia and hypoxia **(B)** was performed to assess an effect of gene silencing on cell proliferation of CIS-treated lung cancer cells. Cell number estimation after autophagy-related genes silencing was performed using Bürker’s chamber **(C)**. Representative western blots showing expression of p62 and LC3B II/I **(D) **in ATGs-silenced cells. Quantification of western blotting results was performed using densitometric analysis and ImageJ software. Data shown as a ratio of a respective protein: p62 **(E) **and LC3B II/I **(F)** to a protein loading control (GAPDH). Each bar represents mean ± SEM. The respective *P-*values was calculated using two-tailed t-test or Mann-Whitney test and a *P*-value < 0.05 was considered statistically significant: **P <* 0.05, ***P <* 0.01, ****P <* 0.001, n ≥ 3.

Supplementary Figure 7 HCQ affects expression of proteins related to autophagy, proliferation and senescence in CIS-treated A549 lung cancer cells and it increases apoptosis of non-senescent cancer cells. All analyses were performed on 11^th^ day of the experiment (according to **Figure 8C**). Quantification of protein expression in HCQ- and CIS-treated cells was performed using densitometric analysis and ImageJ software. Data shown as a ratio of the respective protein: p62 **(A)**, LC3B II/I **(B)**, cyclin B **(C)**, p-cdc2 **(D)**, p-21 **(E)**, and p53 **(F)** to a protein loading control (GAPDH). Distribution of HCQ-treated non-senescent lung cancer cells in subG1 phase of cell cycle was evaluated using PI staining and flow cytometry **(G)**. Cell number estimation of non-senescent cells treated with HCQ was performed using Bürker’s chamber** (H)**. Each bar represents mean ± SEM. The respective *P-*values was calculated using two-tailed t-test or Mann-Whitney test and a *P*-value  < 0.05 was considered statistically significant. **P <* 0.05, ***P <* 0.01, ****p* value < 0.001, #*P <* 0.05, ##*P <* 0.01, ###*P <* 0.001 comparing to hypoxic control, $*P* < 0.05, $$*P <* 0.01, $$$*P <* 0.001 comparing hypoxia to normoxia, n ≥ 3.

Supplementary Figure 8 HCQ effects expression of stem cell-related genes in CIS-treated lung cancer cells. All analyses after HCQ and CIS treatment were performed on 9^th^ day of the experiment (according to **Figure 10A**). Expression of: SOX-2 **(A)**, Nanog **(B)**, ALDH1 **(C)** and OCT-3/4 **(D)** genes was measured using qRT-PCR. GAPDH or PPIA were used as a reference genes. The respective *P-*values was calculated using two-tailed t-test or Mann-Whitney test and a *P*-value  < 0.05 was considered statistically significant. **P <* 0.05, ***P <* 0.01, ****P <* 0.001 comparing to normoxic control, #*P <* 0.05, ##*P <* 0.01, ###*P <* 0.001 comparing to hypoxic control, $*P <* 0.05, $$*P <* 0.01, $$$*P <* 0.001 comparing hypoxia to normoxia, *n* ≥ 3.

## References

[1] Barta JA Powell CA Wisnivesky JP. Global Epidemiology of Lung Cancer. Ann Glob Health (2019) 85(1):1–16. doi: 10.5334/aogh.2419 30741509 PMC6724220

[2] Arbour KC Riely GJ. Systemic Therapy for Locally Advanced and Metastatic Non-Small Cell Lung Cancer: A Review. JAMA (2019) 322(8):764–74. doi: 10.1001/jama.2019.11058 31454018

[3] Duma N Santana-Davila R Molina JR. Non-Small Cell Lung Cancer: Epidemiology, Screening, Diagnosis, and Treatment. Mayo Clin Proc (2019) 94(8):1623–40. doi: 10.1016/j.mayocp.2019.01.013 31378236

[4] Ben Amar J Ben Safta B Zaibi H Dhahri B Ali Baccar M Azzabi S. Prognostic Factors of Advanced Stage non-Small-Cell Lung Cancer. Tunis Med (2016) 94(5):360–7. 27801487

[5] Chen J Huang X Tao C Xiao T Li X Zeng Q. Artemether Attenuates the Progression of Non-Small Cell Lung Cancer by Inducing Apoptosis, Cell Cycle Arrest and Promoting Cellular Senescence. Biol Pharm Bull (2019) 42(10):1720–5. doi: 10.1248/bpb.b19-00391 31378747

[6] Gupta S Silveira DA Mombach JCM. ATM/miR-34a-5p Axis Regulates a P21-Dependent Senescence-Apoptosis Switch in non-Small Cell Lung Cancer: A Boolean Model of G1/S Checkpoint Regulation. FEBS Lett (2020) 594(2):227–39. doi: 10.1002/1873-3468.13615 31545515

[7] Saleh T Bloukh S Carpenter VJ Alwohoush E Bakeer J Darwish S. Therapy-Induced Senescence: An "Old" Friend Becomes the Enemy. Cancers (Basel) (2020) 12(4). doi: 10.3390/cancers12040822 PMC7226427 32235364

[8] Basu A. The Interplay Between Apoptosis and Cellular Senescence: Bcl-2 Family Proteins as Targets for Cancer Therapy. Pharmacol Ther (2021) 25:107943. doi: 10.1016/j.pharmthera.2021.107943 34182005

[9] Ewald JA Desotelle JA Wilding G Jarrard DF. Therapy-Induced Senescence in Cancer. J Natl Cancer Inst (2010) 102(20):1536–46. doi: 10.1093/jnci/djq364 PMC2957429 20858887

[10] Nardella C Clohessy JG Alimonti A Pandolfi PP. Pro-Senescence Therapy for Cancer Treatment. Nat Rev Cancer (2011) 11(7):503–11. doi: 10.1038/nrc3057 21701512

[11] Saleh T Tyutyunyk-Massey L Murray GF Alotaibi MR Kawale AS Elsayed Z. Tumor Cell Escape From Therapy-Induced Senescence. Biochem Pharmacol (2019) 162:202–12. doi: 10.1016/j.bcp.2018.12.013 30576620

[12] Ge H Ke J Xu N Li H Gong J Li X. Dexamethasone Alleviates Pemetrexed-Induced Senescence in Non-Small-Cell Lung Cancer. Food Chem Toxicol (2018) 119:86–97. doi: 10.1016/j.fct.2018.05.025 29753869

[13] Wang G Cheng X Zhang J Liao Y Jia Y Quing C. Possibility of Inducing Tumor Cell Senescence During Therapy. Oncol Lett (2021) 22(1):496. doi: 10.3892/ol.2021.12757 33981358 PMC8108274

[14] Schmitt CA. Cellular Senescence and Cancer Treatment. Biochim Biophys Acta (2007) 1775(1):5–20. doi: 10.1016/j.bbcan.2006.08.005 17027159

[15] Demaria M O’Leary MN Chang J Shao L Liu S Almirah F. Cellular Senescence Promotes Adverse Effects of Chemotherapy and Cancer Relapse. Cancer Discovery (2017) 7(2):165–76. doi: 10.1158/2159-8290.CD-16-0241 PMC5296251 27979832

[16] Was H Barszcz K Czarnecka J Kowalczyk A Bernas T Uzarowska E. Bafilomycin A1 Triggers Proliferative Potential of Senescent Cancer Cells In Vitro and in NOD/SCID Mice. Oncotarget (2017) 8(6):9303–22. doi: 10.18632/oncotarget.14066 PMC5354733 28030837

[17] Was H Czarnecka J Kominek A Bernas T Piwocka K Kaminska B. Some Chemotherapeutics-Treated Colon Cancer Cells Display a Specific Phenotype Being a Combination of Stem-Like and Senescent Cell Features. Cancer Biol Ther (2018) 19(1):63–75. doi: 10.1080/15384047.2017.1385675 29053388 PMC5790359

[18] Telleria CM. Repopulation of Ovarian Cancer Cells After Chemotherapy. Cancer Growth Metastasis (2013) 6:15–21. doi: 10.4137/CGM.S11333 23544004 PMC3611091

[19] Was H Borkowska A Olszewska A Klemba A Marciniak M Synowiec A. Polyploidy Formation in Cancer Cells: How a Trojan Horse is Born. Semin Cancer Biol (2021) 13:S1044-579X(21):00053–5. doi: 10.1016/j.semcancer.2021.03.003 33727077

[20] Mosieniak G Sliwinska MA Alster O Strzeszewska A Sunerland P Piechota M. Polyploidy Formation in Doxorubicin-Treated Cancer Cells Can Favor Escape From Senescence. Neoplasia (2015) 17(12):882–93. doi: 10.1016/j.neo.2015.11.008 PMC4688565 26696370

[21] Erenpreisa J Salmina K Huna A Jackson TR Vazquez-Martin A Cragg MS. The "Virgin Birth", Polyploidy, and the Origin of Cancer. Oncoscience (2015) 2(1):3–14. doi: 10.18632/oncoscience.108 25821840 PMC4341460

[22] Milanovic M Fan DNY Belenki D Däbritz JHM Zhao Z Yu Y. Senescence-Associated Reprogramming Promotes Cancer Stemness. Nature (2018) 553(7686):96–100. doi: 10.1038/nature25167 29258294

[23] Yang L Fang J Chen J. Tumor Cell Senescence Response Produces Aggressive Variants. Cell Death Discovery (2017) 3:17049. doi: 10.1038/cddiscovery.2017.49 28845296 PMC5563524

[24] Niu N Mercado-Uribe I Liu J. Dedifferentiation Into Blastomere-Like Cancer Stem Cells via Formation of Polyploid Giant Cancer Cells. Oncogene (2017) 36(34):4887–900. doi: 10.1038/onc.2017.72 PMC5582213 28436947

[25] Lopez-Sanchez LM Jimenez C Valverde A Hernandez V Penarando J Martinez A. CoCl2, a Mimic of Hypoxia, Induces Formation of Polyploid Giant Cells With Stem Characteristics in Colon Cancer. PloS One (2014) 9(6):e99143. doi: 10.1371/journal.pone.0099143 24932611 PMC4059626

[26] Zhang S Uribe-Mercado I Xing Z Sun B Kuang J Liu J. Generation of Cancer Stem-Like Cells Through the Formation of Polyploid Giant Cancer Cells. Oncogene (2014) 33(1):116–28. doi: 10.1038/onc.2013.96 PMC3844126 23524583

[27] Goehe RW Di X Sharma K Bristol ML Henderson SC Valerie K. The Autophagy-Senescence Connection in Chemotherapy: Must Tumor Cells (Self) Eat Before They Sleep? J Pharmacol Exp Ther (2012) 343(3):763–78. doi: 10.1124/jpet.112.197590 PMC3500537 22927544

[28] Bojko A Staniak K Czarnecka-Herok J Sunderland P Dudkowska M Sliwinska MA. Improved Autophagic Flux in Escapers From Doxorubicin-Induced Senescence/Polyploidy of Breast Cancer Cells. Int J Mol Sci (2020) 21(17). doi: 10.3390/ijms21176084 PMC7504443 32846959

[29] Yan Y Chan X Wang X Zhao Z Hu W Zeng S. The Effects and the Mechanisms of Autophagy on the Cancer-Associated Fibroblasts in Cancer. J Exp Clin Cancer Res (2019) 38(1):171. doi: 10.1186/s13046-019-1172-5 31014370 PMC6480893

[30] Gewirtz DA. Autophagy and Senescence in Cancer Therapy. J Cell Physiol (2014) 229(1):6–9. doi: 10.1002/jcp.24420 23794221

[31] Rajendran P Alzahrani AM Hanieh HN Ashok Kumar S Ben Ammar R Rengarajan T. Autophagy and Senescence: A New Insight in Selected Human Diseases. J Cell Physiol (2019) 234(12):21485–92. doi: 10.1002/jcp.28895 31144309

[32] Kwon Y Kim JW Jeoung JA Kim MS Kang C. Autophagy Is Pro-Senescence When Seen in Close-Up, But Anti-Senescence in Long-Shot. Mol Cells (2017) 40(9):607–12. doi: 10.14348/molcells.2017.0151 PMC5638768 28927262

[33] Kang C Xu Q Martin TD Li MZ Demaria M Aron L. The DNA Damage Response Induces Inflammation and Senescence by Inhibiting Autophagy of GATA4. Science (2015) 349(6255):aaa5612. doi: 10.1126/science.aaa5612 26404840 PMC4942138

[34] Carreau A El Hafny-Rahbi B Matejuk A Grillon C Kieda C. Why is the Partial Oxygen Pressure of Human Tissues a Crucial Parameter? Small Molecules and Hypoxia. J Cell Mol Med (2011) 15(6):1239–53. doi: 10.1111/j.1582-4934.2011.01258.x PMC4373326 21251211

[35] Ye IC Fertig EJ DiGiacomo JW Considine M Godet I Gilkes DM. Molecular Portrait of Hypoxia in Breast Cancer: A Prognostic Signature and Novel HIF-Regulated Genes. Mol Cancer Res (2018) 16(12):1889–901. doi: 10.1158/1541-7786.MCR-18-0345 PMC6279594 30037853

[36] Ye Y Hu Q Chen H Liang K Yuan Y Xiang Y. Characterization of Hypoxia-Associated Molecular Features to Aid Hypoxia-Targeted Therapy. Nat Metab (2019) 1(4):431–44. doi: 10.1038/s42255-019-0045-8 PMC6980239 31984309

[37] Doktorova H Hrabeta J Khalil MA Eckschlager T. Hypoxia-Induced Chemoresistance in Cancer Cells: The Role of Not Only HIF-1. BioMed Pap Med Fac Univ Palacky Olomouc Czech Repub (2015) 159(2):166–77. doi: 10.5507/bp.2015.025 26001024

[38] Jing X Yang F Shao C Wei K Xie M Shen H. Role of Hypoxia in Cancer Therapy by Regulating the Tumor Microenvironment. Mol Cancer (2019) 18(1):157. doi: 10.1186/s12943-019-1089-9 31711497 PMC6844052

[39] Dimri GP Lee X Basile G Acosta M Scott G Roskelley C. A Biomarker That Identifies Senescent Human Cells in Culture and in Aging Skin In Vivo. Proc Natl Acad Sci U.S.A. (1995) 92(20):9363–7. doi: 10.1073/pnas.92.20.9363 PMC40985 7568133

[40] Zhang H. Molecular Signaling and Genetic Pathways of Senescence: Its Role in Tumorigenesis and Aging. J Cell Physiol (2007) 210(3):567–74. doi: 10.1002/jcp.20919 17133363

[41] Xu Y Her C. Inhibition of Topoisomerase (DNA) I (TOP1): DNA Damage Repair and Anticancer Therapy. Biomolecules (2015) 5(3):1652–70. doi: 10.3390/biom5031652 PMC4598769 26287259

[42] Fujita K Kubota Y Ishida H Sasaki Y. Irinotecan, a Key Chemotherapeutic Drug for Metastatic Colorectal Cancer. World J Gastroenterol (2015) 21(43):12234–48. doi: 10.3748/wjg.v21.i43.12234 PMC4649109 26604633

[43] Saoudaoui S Bernard M Cardin GB Malaquin N Christopoulos A Rodier F. mTOR as a Senescence Manipulation Target: A Forked Road. Adv Cancer Res (2021) 150:335–63. doi: 10.1016/bs.acr.2021.02.002 33858600

[44] Blagosklonny MV. Hypoxia, MTOR and Autophagy: Converging on Senescence or Quiescence. Autophagy (2013) 9(2):260–2. doi: 10.4161/auto.22783 PMC3552896 23192222

[45] Patel NH Bloukh S Alwohosh E Alhesa A Saleh T Gewirtz DA. Autophagy and Senescence in Cancer Therapy. Adv Cancer Res (2021) 150:1–74. doi: 10.1016/bs.acr.2021.01.002 33858594

[46] Cao QH Liu F Yang Z Fu X Yang Z Liu Q. Prognostic Value of Autophagy Related Proteins ULK1, Beclin 1, ATG3, ATG5, ATG7, ATG9, ATG10, ATG12, LC3B and P62/SQSTM1 in Gastric Cancer. Am J Transl Res (2016) 8(9):3831–47. PMC5040681 27725863

[47] Ferreira PMP Sousa RWR Ferreira JRO Militão GCG Bezerra DP. Chloroquine and Hydroxychloroquine in Antitumor Therapies Based on Autophagy-Related Mechanisms. Pharmacol Res (2021) 168:105582. doi: 10.1016/j.phrs.2021.105582 33775862

[48] Agnoletto C Caruso C Garofalo C. Heterogeneous Circulating Tumor Cells in Sarcoma: Implication for Clinical Practice. Cancers (Basel) (2021) 13(9). doi: 10.3390/cancers13092189 PMC8124844 34063272

[49] Daniel Y Lelou E Aninat C Corlu A Cabillic F. Interplay Between Metabolism Reprogramming and Epithelial-To-Mesenchymal Transition in Cancer Stem Cells. Cancers (Basel) (2021) 13(8). doi: 10.3390/cancers13081973 PMC8072988 33923958

[50] Ayla S Karahuseyinogluc S. Cancer Stem Cells, Their Microenvironment and Anoikis. Crit Rev Oncog (2019) 24(1):27–34. doi: 10.1615/CritRevOncog.2018029433 31679217

[51] Bhuria V Xing J Scholta T Bui KC Nguyen MLT Malek NP. Hypoxia Induced Sonic Hedgehog Signaling Regulates Cancer Stemness, Epithelial-to-Mesenchymal Transition and Invasion in Cholangiocarcinoma. Exp Cell Res (2019) 385(2):111671. doi: 10.1016/j.yexcr.2019.111671 31634481

[52] Gloushankova NA Zhitnyak IY Rubtsova SN. Role of Epithelial-Mesenchymal Transition in Tumor Progression. Biochem (Mosc) (2018) 83(12):1469–76. doi: 10.1134/S0006297918120052 30878022

[53] Forte E Chimenti I Rosa P Angelini F Pagano F Calogero A. EMT/MET at the Crossroad of Stemness, Regeneration and Oncogenesis: The Ying-Yang Equilibrium Recapitulated in Cell Spheroids. Cancers (Basel) (2017) 9(8). doi: 10.3390/cancers9080098 PMC5575601 28758926

[54] Gao T Li JZ Lu Y Zhang CY Li Q Mao J. The Mechanism Between Epithelial Mesenchymal Transition in Breast Cancer and Hypoxia Microenvironment. BioMed Pharmacother (2016) 80:393–405. doi: 10.1016/j.biopha.2016.02.044 27133080

[55] Marie-Egyptienne DT Lohse I Hill RP. Cancer Stem Cells, the Epithelial to Mesenchymal Transition (EMT) and Radioresistance: Potential Role of Hypoxia. Cancer Lett (2013) 341(1):63–72. doi: 10.1016/j.canlet.2012.11.019 23200673

[56] Hockel M Vaupel P. Tumor Hypoxia: Definitions and Current Clinical, Biologic, and Molecular Aspects. J Natl Cancer Inst (2001) 93(4):266–76. doi: 10.1093/jnci/93.4.266 11181773

[57] Luo W Wang Y. Hypoxia Mediates Tumor Malignancy and Therapy Resistance. Adv Exp Med Biol (2019) 1136:1–18. doi: 10.1007/978-3-030-12734-3_1 31201713

[58] Shin DH Choi YJ Park JW. SIRT1 and AMPK Mediate Hypoxia-Induced Resistance of non-Small Cell Lung Cancers to Cisplatin and Doxorubicin. Cancer Res (2014) 74(1):298–308. doi: 10.1158/0008-5472.CAN-13-2620 24240701

[59] Liu Y Wang X Li W Xu Y Zhuo Y Li M. Oroxylin A Reverses Hypoxia-Induced Cisplatin Resistance Through Inhibiting HIF-1alpha Mediated XPC Transcription. Oncogene (2020) 39(45):6893–905. doi: 10.1038/s41388-020-01474-x 32978517

[60] Pandey N Tyagi G Kaur P Pradhan S Rajam MV Srivastava T. Allicin Overcomes Hypoxia Mediated Cisplatin Resistance in Lung Cancer Cells Through ROS Mediated Cell Death Pathway and by Suppressing Hypoxia Inducible Factors. Cell Physiol Biochem (2020) 54(4):748–66. doi: 10.33594/000000253 32809300

[61] Song X Liu X Chi W Liu Y Wei L Wang X. Hypoxia-Induced Resistance to Cisplatin and Doxorubicin in non-Small Cell Lung Cancer is Inhibited by Silencing of HIF-1alpha Gene. Cancer Chemother Pharmacol (2006) 58(6):776–84. doi: 10.1007/s00280-006-0224-7 16532342

[62] Devarajan N Manjunathan R Ganesan SK. Tumor Hypoxia: The Major Culprit Behind Cisplatin Resistance in Cancer Patients. Crit Rev Oncol Hematol (2021) 162:103327. doi: 10.1016/j.critrevonc.2021.103327 33862250

[63] Rosa P Catacuzzeno L Sforna L Mangino G Carlomagno S Mincione G. BK Channels Blockage Inhibits Hypoxia-Induced Migration and Chemoresistance to Cisplatin in Human Glioblastoma Cells. J Cell Physiol (2018) 233(9):6866–77. doi: 10.1002/jcp.26448 PMC13482417 29319175

[64] Gasiorkiewicz BM Koczurkiewicz-Adamczyk P Piska K Pękala E. Autophagy Modulating Agents as Chemosensitizers for Cisplatin Therapy in Cancer. Invest New Drugs (2021) 39(2):538–63. doi: 10.1007/s10637-020-01032-y PMC7960624 33159673

[65] Rohwer N Dame C Haugstetter A Wiedenmann B Detjen K Schmitt CA. Hypoxia-Inducible Factor 1alpha Determines Gastric Cancer Chemosensitivity via Modulation of P53 and NF-kappaB. PloS One (2010) 5(8):e12038. 10.1371/journal.pone.0012038 20706634 PMC2919384

[66] Lutzkendorf J Wieduwild E Nerger K Lambrecht N Schmoll HJ Müller-Tidow C. Resistance for Genotoxic Damage in Mesenchymal Stromal Cells Is Increased by Hypoxia But Not Generally Dependent on P53-Regulated Cell Cycle Arrest. PloS One (2017) 12(1):e0169921. doi: 10.1371/journal.pone.0169921 28081228 PMC5231334

[67] Song D Beringhs AO Zhuang Z Joshi G Tran TH Claffey KP. Overcoming Hypoxia-Induced Chemoresistance to Cisplatin Through Tumor Oxygenation Monitored by Optical Imaging. Nanotheranostics (2019) 3(2):223–35. doi: 10.7150/ntno.35935 PMC6536783 31183316

[68] Bailly C. Irinotecan: 25 Years of Cancer Treatment. Pharmacol Res (2019) 148:104398. doi: 10.1016/j.phrs.2019.104398 31415916

[69] Chintala S Tóth K Cao S Durrani FA Vaughan MM Jensen RL. Se-Methylselenocysteine Sensitizes Hypoxic Tumor Cells to Irinotecan by Targeting Hypoxia-Inducible Factor 1alpha. Cancer Chemother Pharmacol (2010) 66(5):899–911. doi: 10.1007/s00280-009-1238-8 20066420 PMC2916970

[70] Bhattacharya A Tóth K Mazurchuk R Spernyak JA Slocum HK Pendyala L. Lack of Microvessels in Well-Differentiated Regions of Human Head and Neck Squamous Cell Carcinoma A253 Associated With Functional Magnetic Resonance Imaging Detectable Hypoxia, Limited Drug Delivery, and Resistance to Irinotecan Therapy. Clin Cancer Res (2004) 10(23):8005–17. doi: 10.1158/1078-0432.CCR-04-1306 15585636

[71] Jonchere B Vétillard A Toutain B Lam D Bernard AC Henry C. Irinotecan Treatment and Senescence Failure Promote the Emergence of More Transformed and Invasive Cells That Depend on Anti-Apoptotic Mcl-1. Oncotarget (2015) 6(1):409–26. doi: 10.18632/oncotarget.2774 PMC4381604 25565667

[72] Dai XY Zhuang LH Wang DD Zhou TY Chang LL Gai RH. Nuclear Translocation and Activation of YAP by Hypoxia Contributes to the Chemoresistance of SN38 in Hepatocellular Carcinoma Cells. Oncotarget (2016) 7(6):6933–47. doi: 10.18632/oncotarget.6903 PMC4872759 26771844

[73] Sibaud V Leboeuf NR Roche H Belum VR Gladieff L Deslandres M. Dermatological Adverse Events With Taxane Chemotherapy. Eur J Dermatol (2016) 26(5):427–43. doi: 10.1684/ejd.2016.2833 PMC5526115 27550571

[74] Herbst RS Khuri FR. Mode of Action of Docetaxel - a Basis for Combination With Novel Anticancer Agents. Cancer Treat Rev (2003) 29(5):407–15. doi: 10.1016/S0305-7372(03)00097-5 12972359

[75] Budka JA Ferris MW Capone MJ Hollenhorst PC. Common ELF1 Deletion in Prostate Cancer Bolsters Oncogenic ETS Function, Inhibits Senescence and Promotes Docetaxel Resistance. Genes Cancer (2018) 9(5-6):198–214. doi: 10.18632/genesandcancer.182 30603056 PMC6305106

[76] Simova J Sapega O Imrichova T Stepanek I Kyjacova L Mikyskova R. Tumor Growth Accelerated by Chemotherapy-Induced Senescent Cells is Suppressed by Treatment With IL-12 Producing Cellular Vaccines. Oncotarget (2016) 7(34):54952–64. doi: 10.18632/oncotarget.10712 PMC5342393 27448982

[77] Wang L Fan J Yan CY Ling R Yun J. Activation of Hypoxia-Inducible Factor-1alpha by Prolonged In Vivo Hyperinsulinemia Treatment Potentiates Cancerous Progression in Estrogen Receptor-Positive Breast Cancer Cells. Biochem Biophys Res Commun (2017) 491(2):545–51. doi: 10.1016/j.bbrc.2017.03.128 28351619

[78] Lv Y Zhao S Han J Zheng L Yang Z Zhao L. Hypoxia-Inducible Factor-1alpha Induces Multidrug Resistance Protein in Colon Cancer. Onco Targets Ther (2015) 8:1941–8. doi: 10.2147/OTT.S82835 PMC4524588 26251616

[79] Samanta D Gilkes DM Chaturvedi P Xiang L Semenza GL. Hypoxia-Inducible Factors are Required for Chemotherapy Resistance of Breast Cancer Stem Cells. Proc Natl Acad Sci U.S.A. (2014) 111(50):E5429–38. doi: 10.1073/pnas.1421438111 PMC4273385 25453096

[80] Qian J Shen S Chen W Chen N. Propofol Reversed Hypoxia-Induced Docetaxel Resistance in Prostate Cancer Cells by Preventing Epithelial-Mesenchymal Transition by Inhibiting Hypoxia-Inducible Factor 1alpha. BioMed Res Int (2018) 2018:4174232. doi: 10.1155/2018/4174232 29568752 PMC5820676

[81] Peng XH Karna P Cao Z Jiang BH Zhou M Yang L. Cross-Talk Between Epidermal Growth Factor Receptor and Hypoxia-Inducible Factor-1alpha Signal Pathways Increases Resistance to Apoptosis by Up-Regulating Survivin Gene Expression. J Biol Chem (2006) 281(36):25903–14. doi: 10.1074/jbc.M603414200 PMC3132567 16847054

[82] Wang Q Wu PC Roberson RS Luk BV Ivanova I Chu E. Survivin and Escaping in Therapy-Induced Cellular Senescence. Int J Cancer (2011) 128(7):1546–58. doi: 10.1002/ijc.25482 PMC3000873 20503268

[83] Poklepovic A Gewirtz DA. Outcome of Early Clinical Trials of the Combination of Hydroxychloroquine With Chemotherapy in Cancer. Autophagy (2014) 10(8):1478–80. doi: 10.4161/auto.29428 PMC4203528 24991829

[84] Zeh HJ Bahary N Boone BA Singhi AD Miller-Ocuin JL Normolle DP. A Randomized Phase II Preoperative Study of Autophagy Inhibition With High-Dose Hydroxychloroquine and Gemcitabine/Nab-Paclitaxel in Pancreatic Cancer Patients. Clin Cancer Res (2020) 26(13):3126–34. doi: 10.1158/1078-0432.CCR-19-4042 PMC8086597 32156749

[85] Xu R Ji Z Xu C Zhu J. The Clinical Value of Using Chloroquine or Hydroxychloroquine as Autophagy Inhibitors in the Treatment of Cancers: A Systematic Review and Meta-Analysis. Med (Baltimore) (2018) 97(46):e12912. doi: 10.1097/MD.0000000000012912 PMC6257684 30431566

[86] Wu HM Jiang ZF Ding PS Shao LJ Liu RY. Hypoxia-Induced Autophagy Mediates Cisplatin Resistance in Lung Cancer Cells. Sci Rep (2015) 5:12291. doi: 10.1038/srep12291 26201611 PMC4511870

[87] Song J Qu Z Guo X Zhao Q Zhao X Gao L. Hypoxia-Induced Autophagy Contributes to the Chemoresistance of Hepatocellular Carcinoma Cells. Autophagy (2009) 5(8):1131–44. doi: 10.4161/auto.5.8.9996 19786832

[88] Lee JG Shin JH Shim HS Lee CY Kim DJ Kim YS. Autophagy Contributes to the Chemo-Resistance of Non-Small Cell Lung Cancer in Hypoxic Conditions. Respir Res (2015) 16:138. doi: 10.1186/s12931-015-0285-4 26553068 PMC4640373

[89] Galluzzi L Senovilla L Vitale I Michels J Martins I Kepp O. Molecular Mechanisms of Cisplatin Resistance. Oncogene (2012) 31(15):1869–83. doi: 10.1038/onc.2011.384 21892204

[90] Silva MM Rocha CRR Kinker GS Pelegrini AL Menck CFM. The Balance Between NRF2/GSH Antioxidant Mediated Pathway and DNA Repair Modulates Cisplatin Resistance in Lung Cancer Cells. Sci Rep (2019) 9(1):17639. doi: 10.1038/s41598-019-54065-6 31776385 PMC6881285

[91] Saleh T Tyutyunyk-Massey L Patel NH Cudjoe EK Jr Alotaibi M Gewirtz DA. Studies of Non-Protective Autophagy Provide Evidence That Recovery From Therapy-Induced Senescence is Independent of Early Autophagy. Int J Mol Sci (2020) 21(4). doi: 10.3390/ijms21041427 PMC7073138 32093197

[92] Wakita M Takahashi A Sano O Loo TM Imai Y Narukawa M. A BET Family Protein Degrader Provokes Senolysis by Targeting NHEJ and Autophagy in Senescent Cells. Nat Commun (2020) 11(1):1935. doi: 10.1038/s41467-020-15719-6 32321921 PMC7176673

[93] Bartoszewski R Moszyńska A Serocki M Cabaj A Polten A Ochocka R. Primary Endothelial Cell-Specific Regulation of Hypoxia-Inducible Factor (HIF)-1 and HIF-2 and Their Target Gene Expression Profiles During Hypoxia. FASEB J (2019) 33(7):7929–41. doi: 10.1096/fj.201802650RR PMC6593883 30917010

[94] Saint-Martin A Martínez-Ríos J Castañeda-Patlán MC Sarabia-Sánchez MA Tejeda-Muñoz N Chinney-Herrera A. Functional Interaction of Hypoxia-Inducible Factor 2-Alpha and Autophagy Mediates Drug Resistance in Colon Cancer Cells. Cancers (Basel) (2019) 11(6). doi: 10.3390/cancers11060755 PMC6627604 31151160

[95] Gener P Seras-Franzoso J Callejo PG Andrade F Rafael D Martínez F. Dynamism, Sensitivity, and Consequences of Mesenchymal and Stem-Like Phenotype of Cancer Cells. Stem Cells Int (2018) 2018:4516454. doi: 10.1155/2018/4516454 30405720 PMC6199882

[96] Lu W Kang Y. Epithelial-Mesenchymal Plasticity in Cancer Progression and Metastasis. Dev Cell (2019) 49(3):361–74. doi: 10.1016/j.devcel.2019.04.010 PMC6506183 31063755

[97] Ivanov A Pawlikowski J Manoharan I van Tuyn J Nelson DM Rai TS. Lysosome-Mediated Processing of Chromatin in Senescence. J Cell Biol (2013) 202(1):129–43. doi: 10.1083/jcb.201212110 PMC3704985 23816621

[98] Erenpreisa J Salmina K Huna A Kosmacek EA Cragg MS Ianzini F. Polyploid Tumour Cells Elicit Paradiploid Progeny Through Depolyploidizing Divisions and Regulated Autophagic Degradation. Cell Biol Int (2011) 35(7):687–95. doi: 10.1042/CBI20100762 21250945

[99] Abdul Rahim SA Dirkse A Oudin A Schuster A Bohler J Barthelemy V. Regulation of Hypoxia-Induced Autophagy in Glioblastoma Involves ATG9A. Br J Cancer (2017) 117(6):813–25. doi: 10.1038/bjc.2017.263 PMC5590001 28797031

