# Supplementary figures and images for: Escape From Cisplatin-Induced Senescence of Hypoxic Lung Cancer Cells Can Be Overcome by Hydroxychloroquine
Source: Front Oncol. 2022 Jan 21;11:738385. doi: 10.3389/fonc.2021.738385 (PMC8813758; doi:10.3389/fonc.2021.738385)

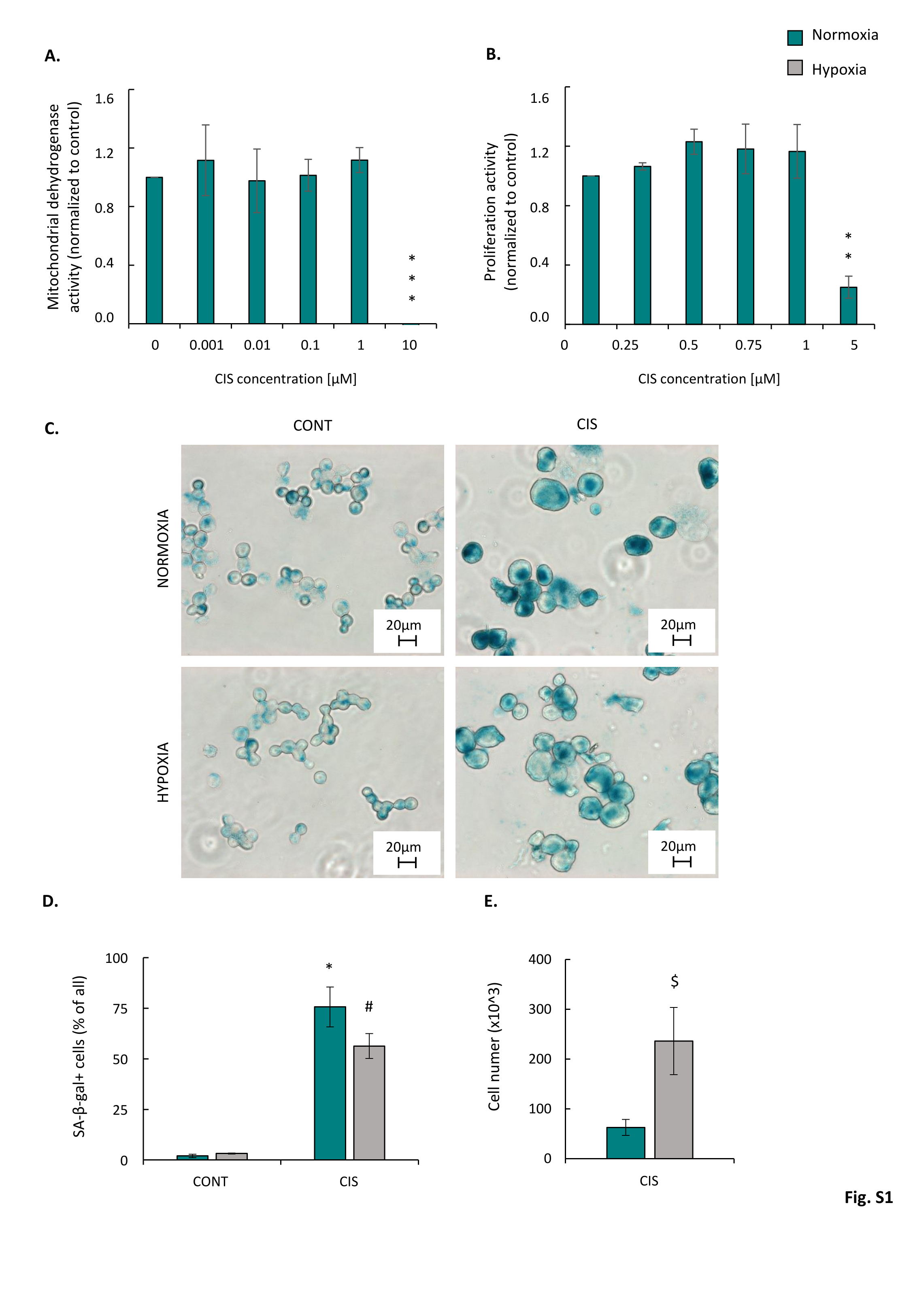

Supplement: Supplementary Figure 1 — Hypoxia reduces markers of senescence and increases proliferation of CIS-treated murine LLC1 lung cancer cells. All analyses of CIS-treated murine LLC1 lung cancer cells were performed on 11th day of the experiment (according to Figure 1A ). MDH test in normoxia (A) was performed for assessment of CIS effect on cell metabolism. BrdU incorporation test in normoxia (B) was performed to assess CIS effects on cell proliferation. Detection and quantification of senescent cells using SA-β-gal staining was performed on cytospined LLC1 cells. Representative photos for cells were acquired using light microscopy: original magnification - 400x, scale bar - 20 µm (C). Quantification of SA-β-gal positive cells was showed as a percentage of positive cells (D). Cell numbers were determined using a Bürker’s chamber (E). Each bar represents mean ± SEM. The respective P-values were calculated using two-tailed t-test or Mann-Whitney test and a P-value < 0.05 was considered statistically significant. *P < 0.05, **P < 0.01, ***P < 0.001 comparing to normoxic control, #P < 0.05, ##P < 0.01, ###P < 0.001 comparing to hypoxic control, $P < 0.05, $$P < 0.01, $$$P < 0.001 comparing hypoxia to normoxia, n ≥ 3. [file Image_1.tiff]

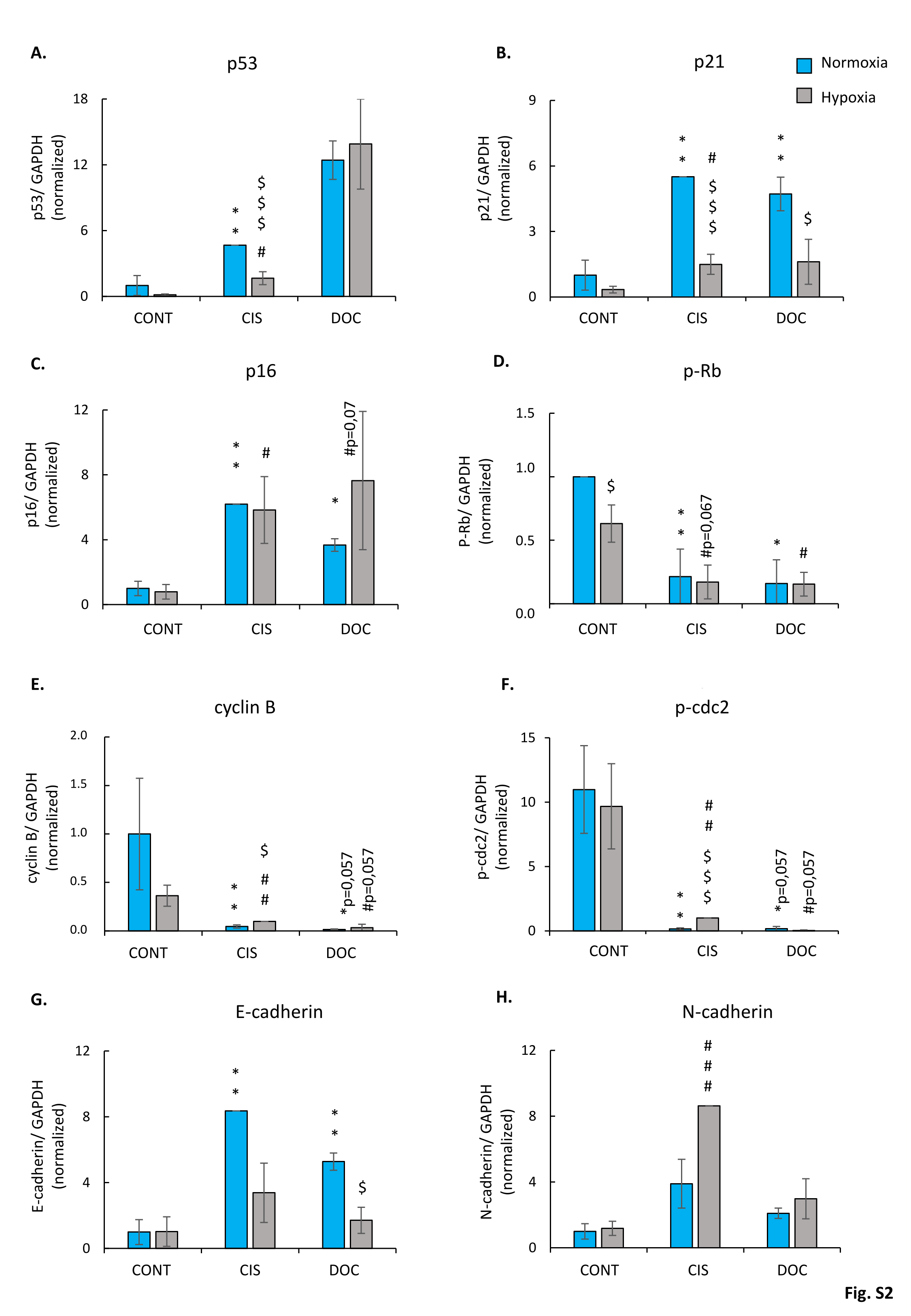

Supplement: Supplementary Figure 2 — Evaluation of protein expression related with senescence, proliferation and EMT in CIS- or DOC-treated A549 lung cancer cells. All analyses after CIS treatment were performed on 11th day of the experiment (according to Figure 1A ). Quantification of western blotting results was performed using densitometric analysis and ImageJ software. Data shown as a ratio of the respective protein to a loading control (GAPDH): p53 (A), p21 (B), p16 (C), pRb (D), cyclin B (E), p-cdc2 (F), E-cadherin (G) and N-cadherin (H). Each bar represents mean ± SEM. The respective P-values were calculated using two-tailed t-test or Mann-Whitney test and a P-value < 0.05 was considered statistically significant. *P < 0.05, **P < 0.01, ***P < 0.001 comparing to normoxic control, #P < 0.05, ##P < 0.01, ###P < 0.001 comparing to hypoxic control, $p value < 0.05, $$P < 0.01, $$$P < 0.001 comparing hypoxia to normoxia, n ≥ 3. [file Image_2.tiff]

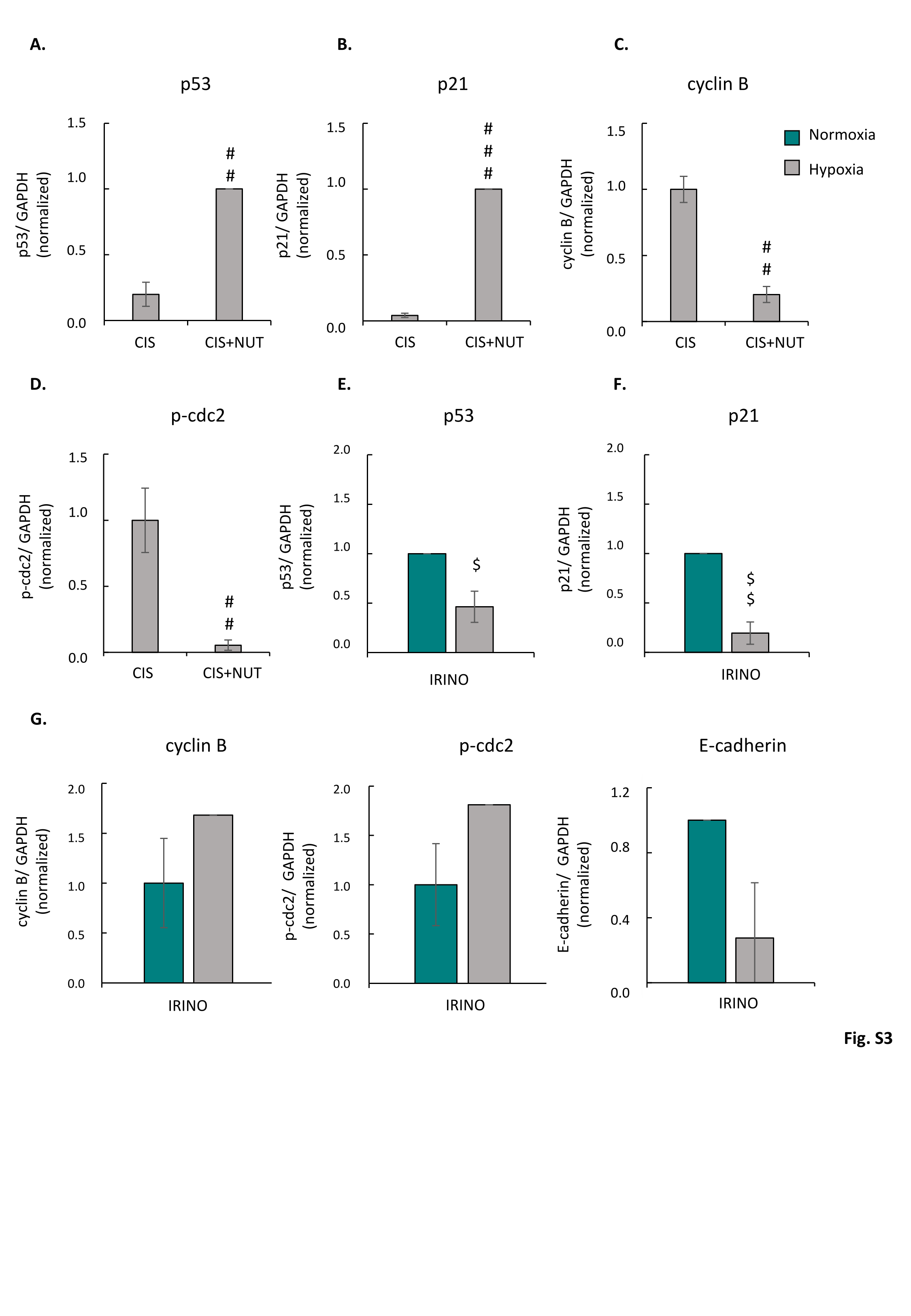

Supplement: Supplementary Figure 3 — Evaluation of expression of proteins related with senescence, proliferation and EMT in NUT- and CIS-treated A549 lung cancer or IRINO-treated HCT116 colon cancer cells. All analyses were performed on 11th day of the experiment (according to Figure 1A ). Quantification of western blotting results was performed using densitometric analysis and ImageJ software. Data shown as a ratio of the respective protein to a loading control (GAPDH). In NUT+CIS experiments: p53 (A), p21 (B), cyclin B (C), p-cdc2 (D), E-cadherin (E) and in IRINO experiments: p53 (E), p21 (F), cyclin B (G), p-cdc2 (H) and E-cadherin (I). Each bar represents mean ± SEM. The respective P-values were calculated using two-tailed t-test or Mann-Whitney test and a P-value < 0.05 was considered statistically significant. $P < 0.05, $$P < 0.01, $$$p value < 0.001 comparing hypoxia to normoxia, n ≥ 3. [file Image_3.tiff]

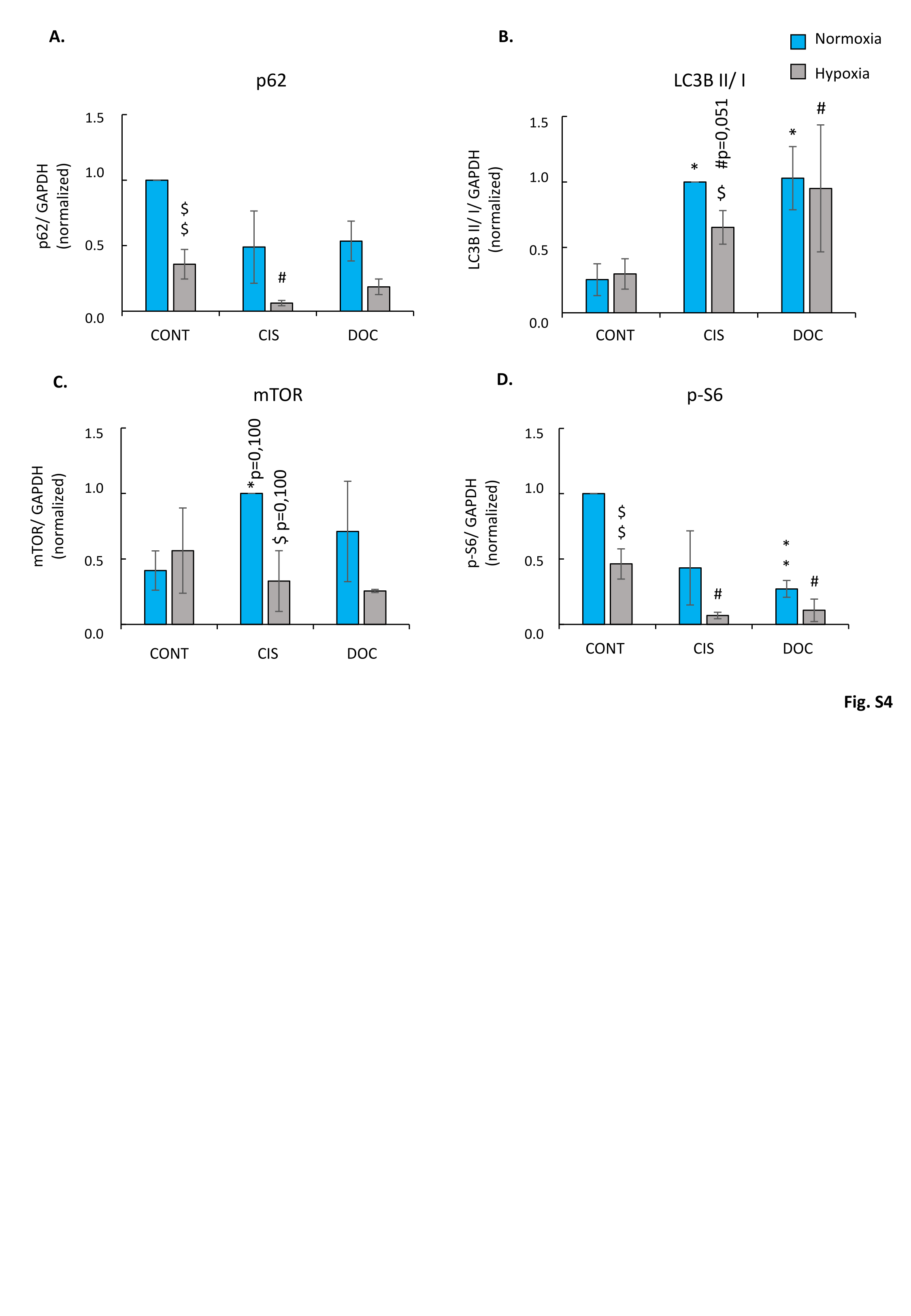

Supplement: Supplementary Figure 4 — Evaluation of expression of proteins associated with autophagy as well as mTOR signaling pathway in CIS- and DOC-treated A549 lung cancer cells. All analyses were performed on 11th day of the experiment (according to Figure 1A ). Quantification of western blotting results was performed using densitometric analysis and ImageJ software. Data shown as a ratio of the respective protein: p62 (A), LC3B II/I (B), mTOR (C) and p-S6 (D) to a loading control (GAPDH). Each bar represents mean ± SEM. The respective P-values was calculated using two-tailed t-test or Mann-Whitney test and a P-value < 0.05 was considered statistically significant. *P < 0.05, **P < 0.01, ***p value < 0.001, #P < 0.05, ##P < 0.01, ###P < 0.001 comparing to hypoxic control, $P < 0.05, $$P < 0.01, $$$P < 0.001 comparing hypoxia to normoxia, n ≥ 3. [file Image_4.tiff]

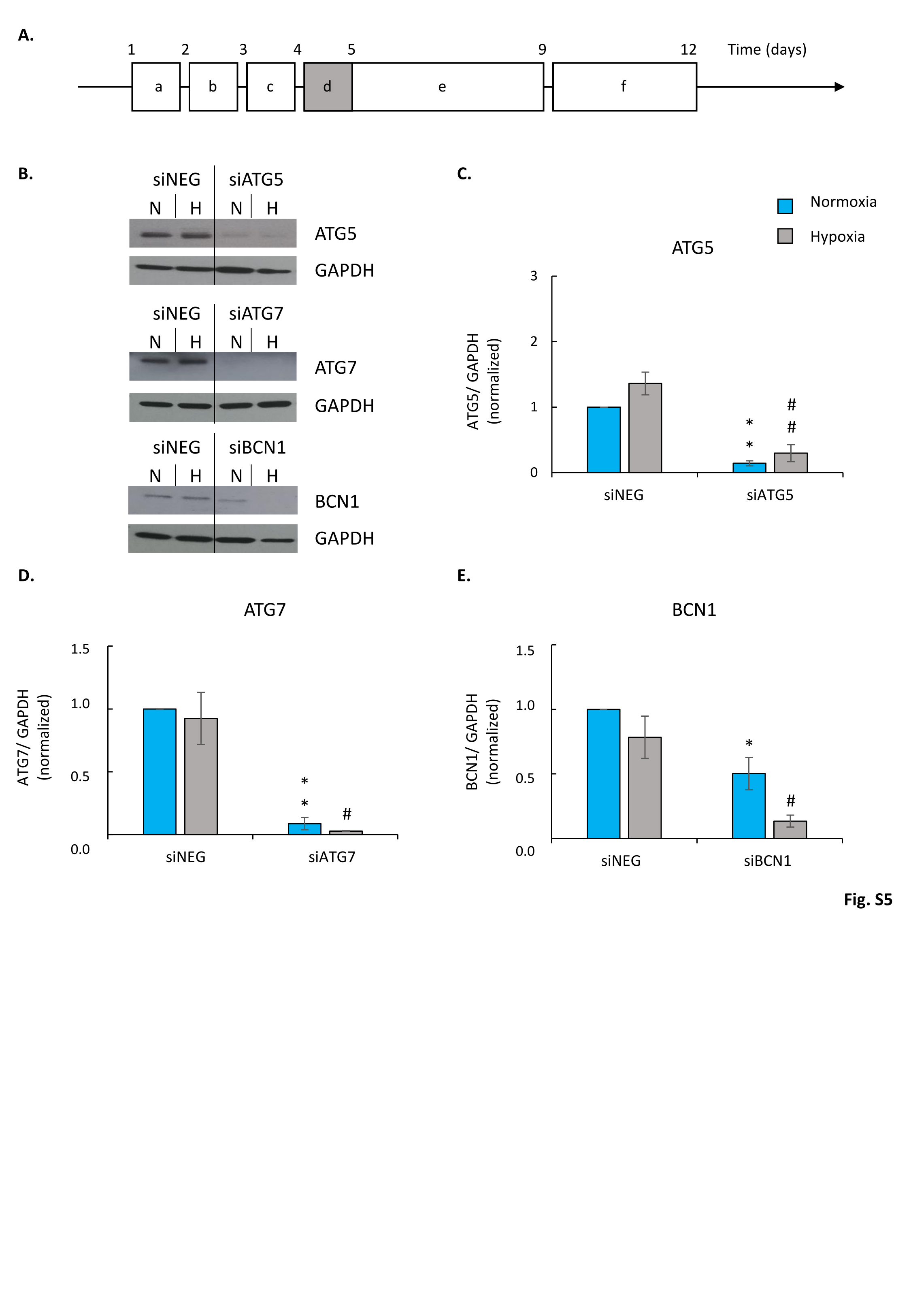

Supplement: Supplementary Figure 5 — Evaluation of efficacy of silencing autophagy-related genes with siRNAs in CIS-treated lung cancer cells. All analyses were performed on 12th day of the experiment (according to A). Scheme of the experiment (5A). Cells were grown in normoxic condition (~19% O2) for 24 hours (a). Then, some plates or flasks were transferred to the hypoxic chamber and the medium was changed (b). Then, autophagy-related gene encoding: BCN1, ATG5 and ATG7 were silenced using siRNAs (c). After next 24 hours the cells were treated with CIS for 24 hours (d) and subsequently cultured in drug-free medium for 4 days (e). Next, medium was changed and cells were incubated for 3 days (f). Western blots with GAPDH as a loading control were performed to confirm ATG5, ATG7 or BCN gene silencing (B). Quantification of western blotting results was performed using densitometric analysis and ImageJ software. Data shown as a ratio of a respective protein: ATG5 (C), ATG7 (D) or BCN1 (E) to a loading control (GAPDH). Each bar represents mean ± SEM. The respective P-values was calculated using two-tailed t-test or Mann-Whitney test and a P-value < 0.05 was considered statistically significant. *P < 0.05, **P < 0.01, ***p value < 0.001, #P < 0.05, ##P < 0.01, ###P < 0.001 comparing to hypoxic control, $P < 0.05, $$P < 0.01, $$$P < 0.001 comparing hypoxia to normoxia, n ≥ 3. [file Image_5.tiff]

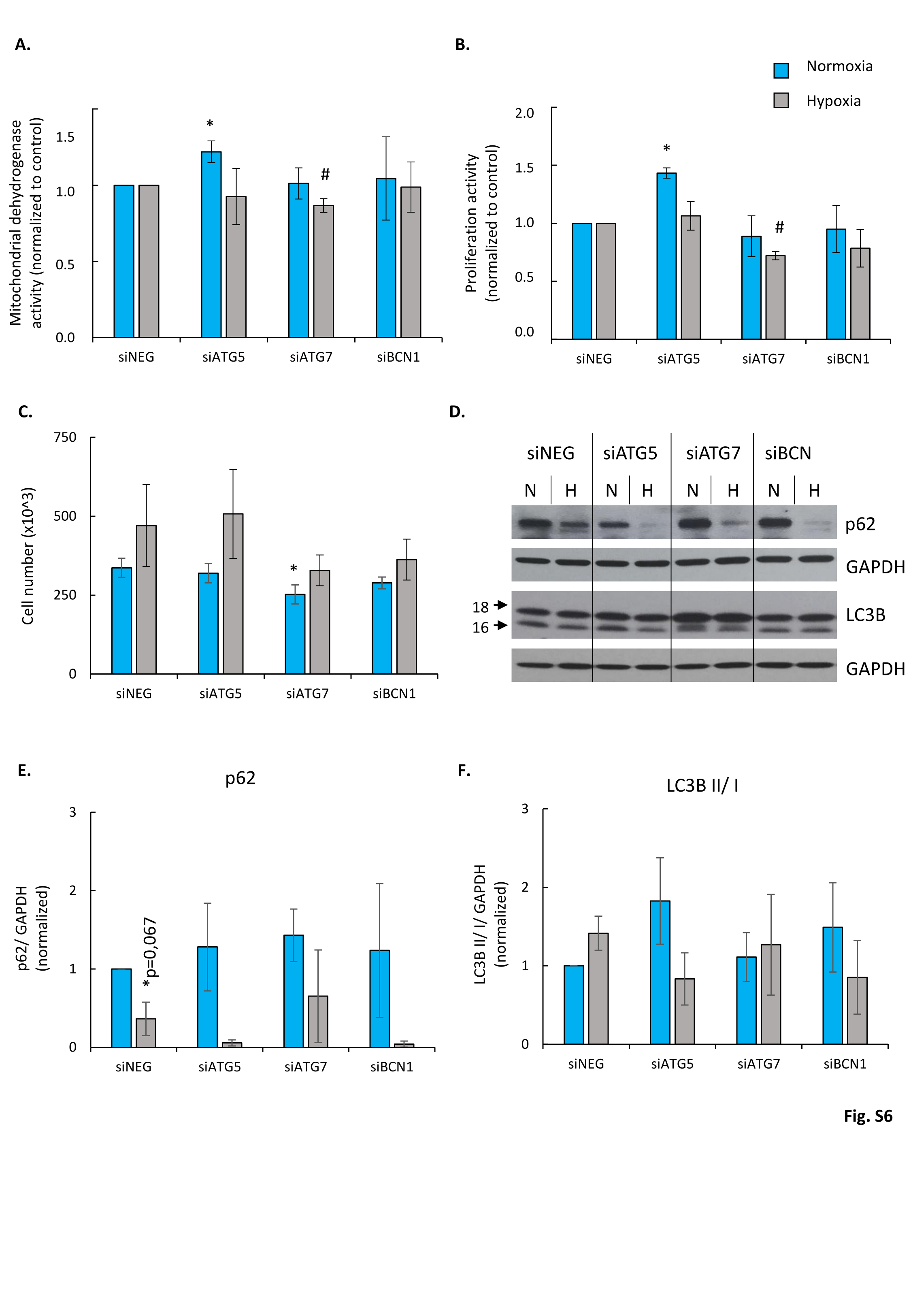

Supplement: Supplementary Figure 6 — Effect of BCN1-, ATG5- and ATG7-silencing on metabolism, proliferation and autophagy of CIS-treated lung cancer cells. All analyses were performed on 12th day of the experiment (according to Supplementary Figure 5A ). MDH test in normoxia or hypoxia (A) was performed to assess an effect of gene silencing on the cell metabolism of CIS-treated lung cancer cells. BrdU incorporation test in normoxia and hypoxia (B) was performed to assess an effect of gene silencing on cell proliferation of CIS-treated lung cancer cells. Cell number estimation after autophagy-related genes silencing was performed using Bürker’s chamber (C). Representative western blots showing expression of p62 and LC3B II/I (D) in ATGs-silenced cells. Quantification of western blotting results was performed using densitometric analysis and ImageJ software. Data shown as a ratio of a respective protein: p62 (E) and LC3B II/I (F) to a protein loading control (GAPDH). Each bar represents mean ± SEM. The respective P-values was calculated using two-tailed t-test or Mann-Whitney test and a P-value < 0.05 was considered statistically significant: *P < 0.05, **P < 0.01, ***P < 0.001, n ≥ 3. [file Image_6.tiff]

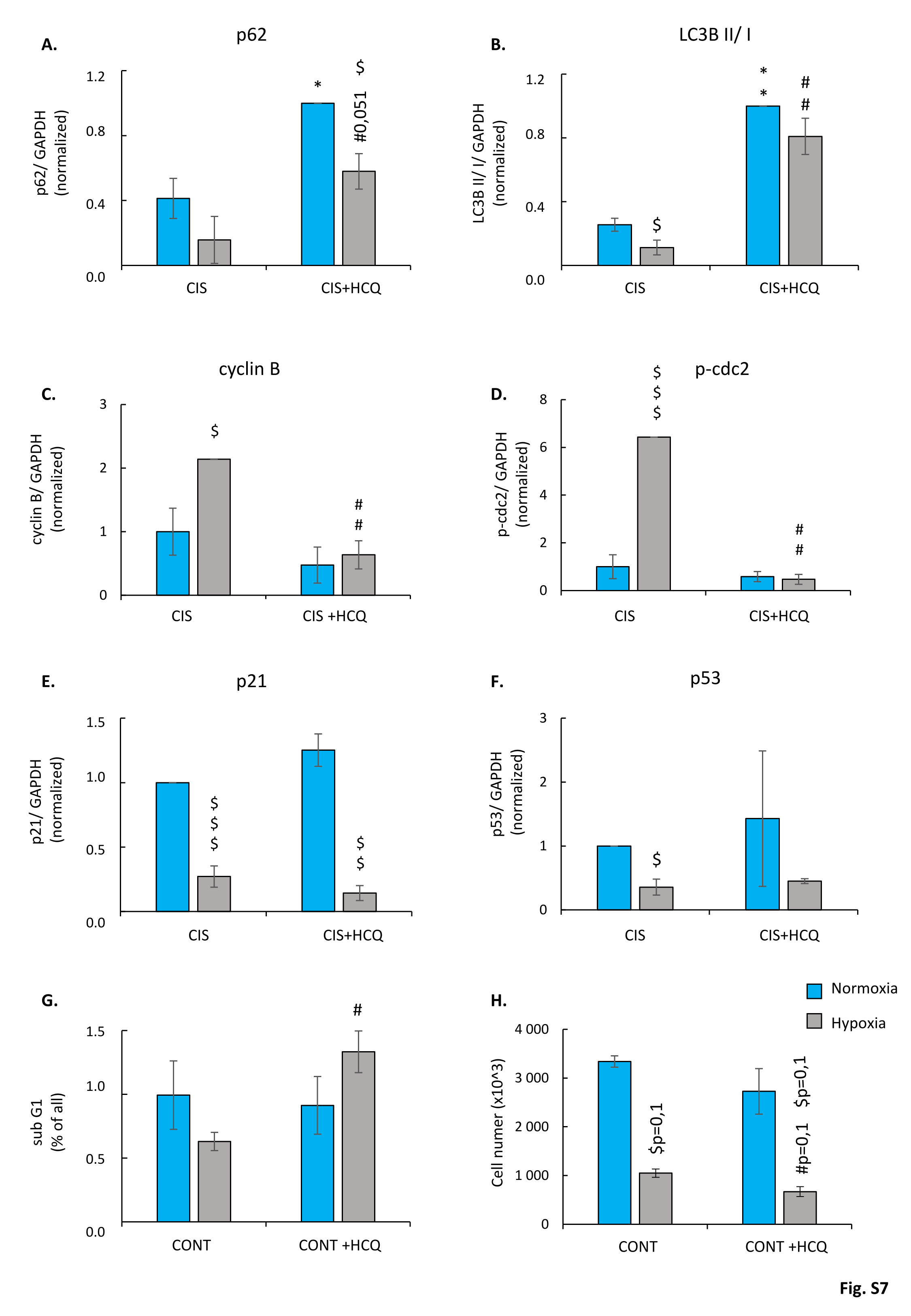

Supplement: Supplementary Figure 7 — HCQ affects expression of proteins related to autophagy, proliferation and senescence in CIS-treated A549 lung cancer cells and it increases apoptosis of non-senescent cancer cells. All analyses were performed on 11th day of the experiment (according to Figure 8C ). Quantification of protein expression in HCQ- and CIS-treated cells was performed using densitometric analysis and ImageJ software. Data shown as a ratio of the respective protein: p62 (A), LC3B II/I (B), cyclin B (C), p-cdc2 (D), p-21 (E), and p53 (F) to a protein loading control (GAPDH). Distribution of HCQ-treated non-senescent lung cancer cells in subG1 phase of cell cycle was evaluated using PI staining and flow cytometry (G). Cell number estimation of non-senescent cells treated with HCQ was performed using Bürker’s chamber (H). Each bar represents mean ± SEM. The respective P-values was calculated using two-tailed t-test or Mann-Whitney test and a P-value < 0.05 was considered statistically significant. *P < 0.05, **P < 0.01, ***p value < 0.001, #P < 0.05, ##P < 0.01, ###P < 0.001 comparing to hypoxic control, $P < 0.05, $$P < 0.01, $$$P < 0.001 comparing hypoxia to normoxia, n ≥ 3. [file Image_7.tiff]

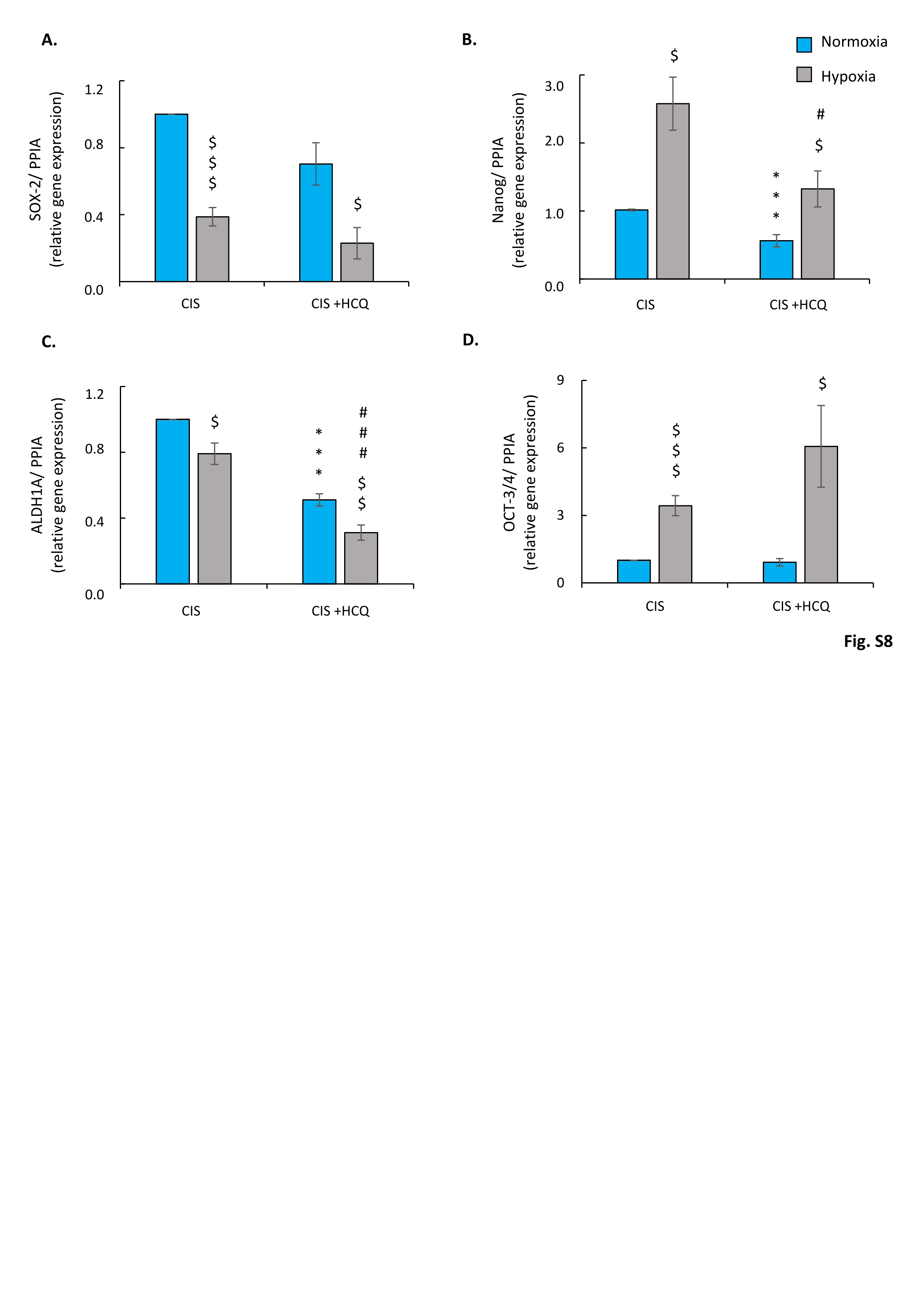

Supplement: Supplementary Figure 8 — HCQ effects expression of stem cell-related genes in CIS-treated lung cancer cells. All analyses after HCQ and CIS treatment were performed on 9th day of the experiment (according to Figure 10A ). Expression of: SOX-2 (A), Nanog (B), ALDH1 (C) and OCT-3/4 (D) genes was measured using qRT-PCR. GAPDH or PPIA were used as a reference genes. The respective P-values was calculated using two-tailed t-test or Mann-Whitney test and a P-value < 0.05 was considered statistically significant. *P < 0.05, **P < 0.01, ***P < 0.001 comparing to normoxic control, #P < 0.05, ##P < 0.01, ###P < 0.001 comparing to hypoxic control, $P < 0.05, $$P < 0.01, $$$P < 0.001 comparing hypoxia to normoxia, n ≥ 3. [file Image_8.tiff]

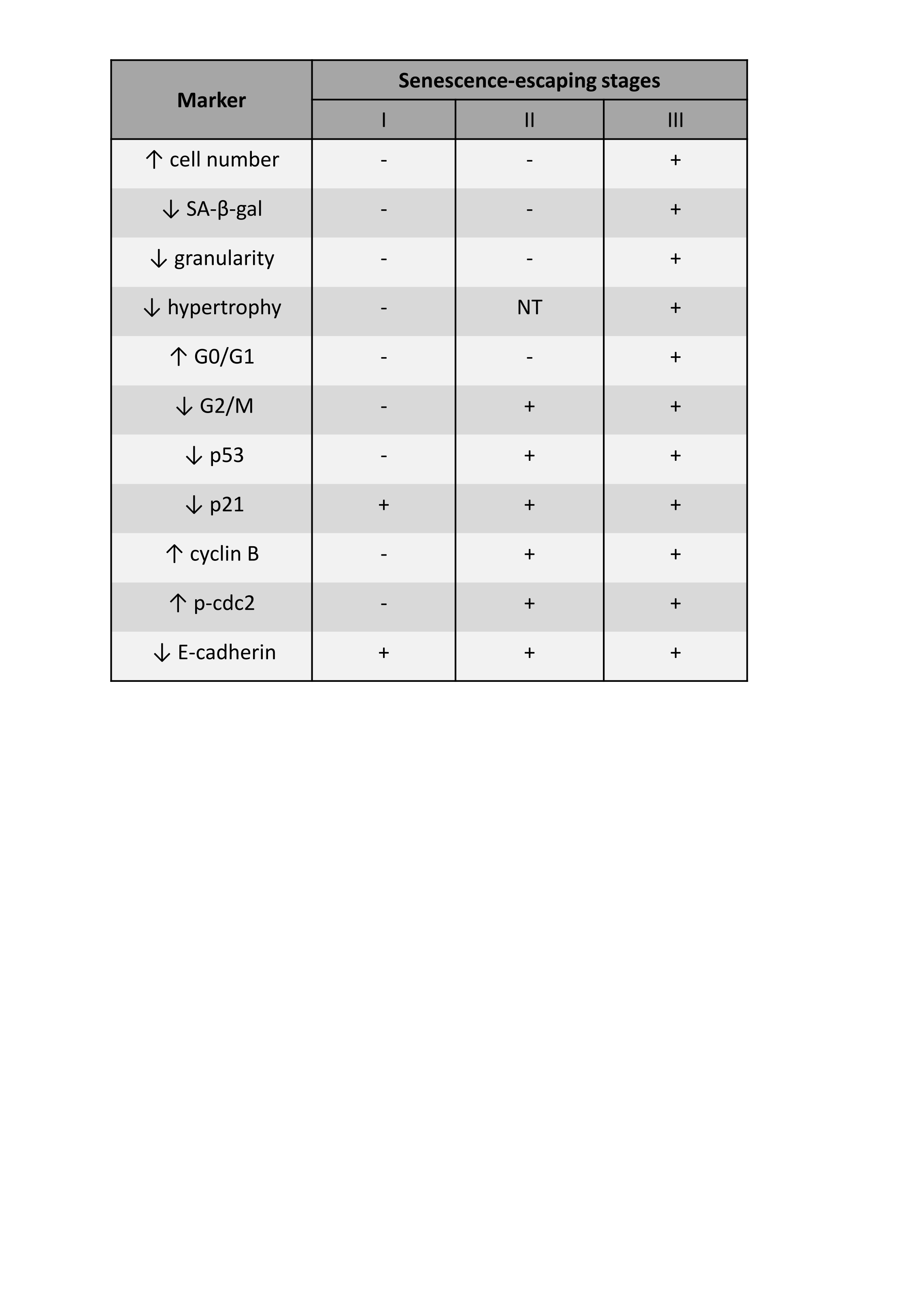

Supplement: Supplementary file 9 [file Image_9.tiff]

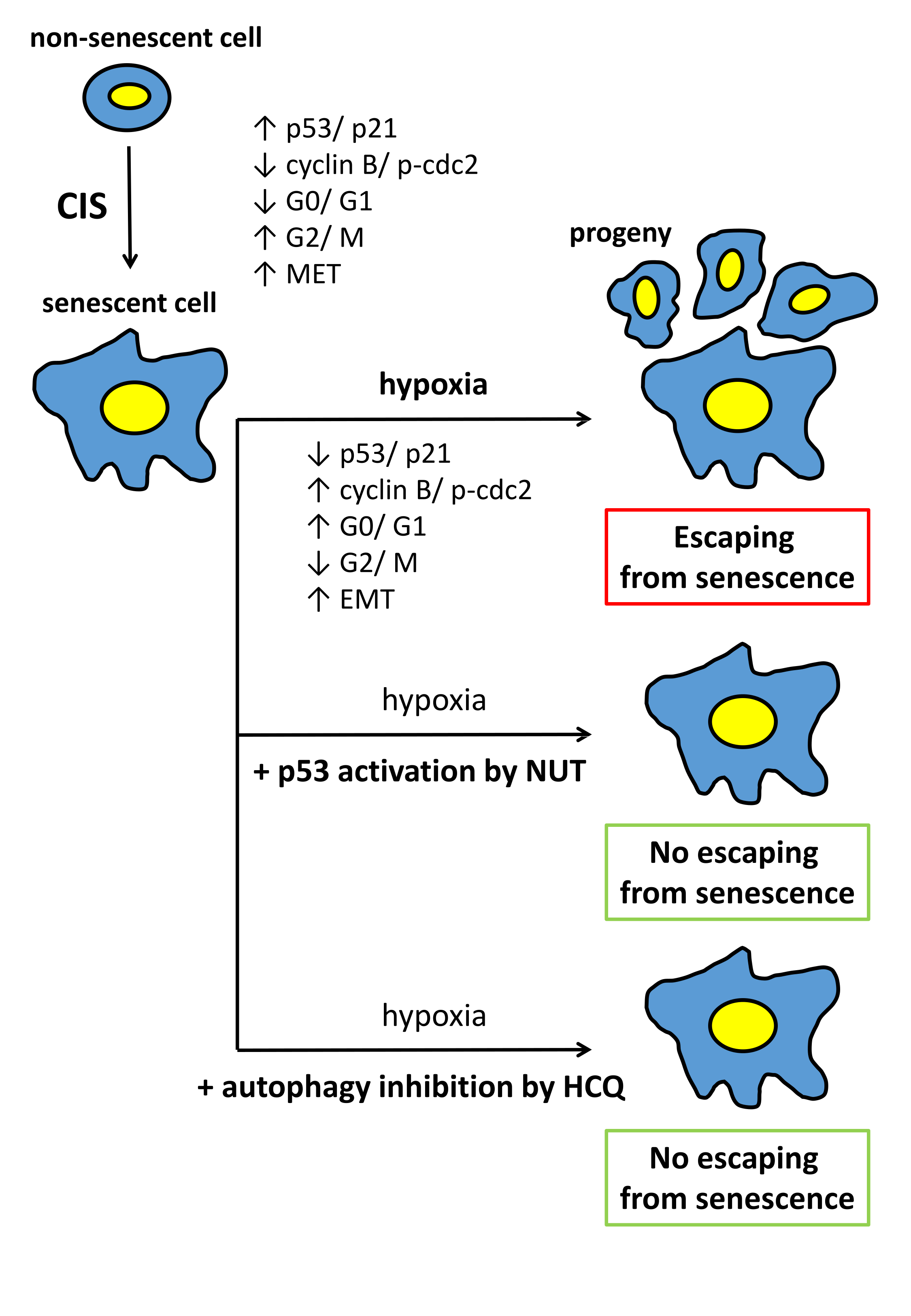

Supplement: Supplementary file 10 [file Image_10.tiff]
